# Microwave-assisted synthesis of iridium oxide and palladium nanoparticles supported on a nitrogen-rich covalent triazine framework as superior electrocatalysts for the hydrogen evolution and oxygen reduction reaction

**DOI:** 10.3389/fchem.2022.945261

**Published:** 2022-07-26

**Authors:** Lars Rademacher, Thi Hai Yen Beglau, Tobias Heinen, Juri Barthel, Christoph Janiak

**Affiliations:** ^1^ Institut für Anorganische Chemie und Strukturchemie, Heinrich-Heine-Universität Düsseldorf, Düsseldorf, Germany; ^2^ Ernst Ruska-Zentrum für Mikroskopie und Spektroskopie mit Elektronen, Forschungszentrum Jülich GmbH, Jülich, Germany

**Keywords:** iridium oxide, palladium, nanoparticles, ionic liquid, propylene carbonate, covalent triazine framework, hydrogen evolution reaction, oxygen reduction reaction

## Abstract

Iridium oxide (IrO_x_-NP) and palladium nanoparticles (Pd-NP) were supported on a 2,6-dicyanopyridine-based covalent-triazine framework (DCP-CTF) by energy-saving and sustainable microwave-assisted thermal decomposition reactions in propylene carbonate and in the ionic liquid [BMIm][NTf_2_]. Transmission electron microscopy (TEM), scanning electron microscopy (SEM), and X-ray photoelectron spectroscopy (XPS) confirm well-distributed NPs with sizes from 2 to 13 nm stabilized on the CTF particles. Metal contents between 10 and 41 wt% were determined by flame atomic absorption spectroscopy (AAS). Nitrogen sorption measurements of the metal-loaded CTFs revealed Brunauer–Emmett–Teller (BET) surface areas between 904 and 1353 m^2^ g^−1^. The composites show superior performance toward the hydrogen evolution reaction (HER) with low overpotentials from 47 to 325 mV and toward the oxygen reduction reaction (ORR) with high half-wave potentials between 810 and 872 mV. IrO_x_ samples in particular show high performances toward HER while the Pd samples show better performance toward ORR. In both reactions, electrocatalysts can compete with the high performance of Pt/C. Exemplary cyclic voltammetry durability tests with 1000 cycles and subsequent TEM analyses show good long-term stability of the materials. The results demonstrate the promising synergistic effects of NP-decorated CTF materials, resulting in a high electrocatalytic activity and stability.

## 1 Introduction

The hydrogen evolution reaction (HER) and the oxygen reduction reaction (ORR) are crucial processes for achieving a sustainable and CO_2_-free energy supply in many industrial sectors (Gretz et al., 1991; Shao et al., 2016; Dubouis and Grimaud, 2019; Zhu et al., 2020; Dong et al., 2021; Rahman et al., 2021). Many studies have focused on noble metal-based electrocatalysts, mainly platinum, due to their superior electrocatalytic activity toward both reactions (Zheng et al., 2014; Liu et al., 2017). Due to the high costs of noble metals, their nanoparticular state is an area of active research. Nanoparticles (NPs) provide a high surface-to-volume ratio so that the mass-based catalytic properties improve through the high fraction of surface atoms (Janiak, 2013; Wang et al., 2018; Zeng and Li, 2015; Jeevanandam et al., 2018).

However, the thermodynamically favored agglomeration of NPs requires the use of molecular stabilizers. One extensively studied class of NP stabilizers is ionic liquids (ILs). ILs consist of weakly coordinating ions that prevent the agglomeration of metal nanoparticles (M-NP) by electrostatic and steric interactions, also known as electrostatic interactions (Janiak, 2013; Fredlake et al., 2004; Wegner and Janiak, 2017). Alternatively, propylene carbonate (PC) also functions as a stabilizing agent in the synthesis of M-NPs (Vollmer et al., 2012; Esteban et al., 2015a; Siebels et al., 2018). PC is of interest due to its low toxicity and high sustainability contrary to other organic solvents, giving PC the designation as a *green* solvent (Schäffner et al., 2009; Alder et al., 2016; Millán et al., 2020). Furthermore, both ILs and PC have negligible vapor pressure and are highly dipolar (Schütte et al., 2014). These combined properties are essential factors for an efficient, additive-free, and safe synthesis of metal NP by a fast microwave-induced synthesis, potentially superior to other NP preparation methods using conventional heating or further additives (Bilecka and Niederberger, 2010; Marquardt et al., 2011; Vollmer et al., 2012; Siebels et al., 2018).

In addition, carbon materials with high porosity and electrical conductivity are often used as stabilizing support for M-NPs (Liu Y. et al., 2019; Woitassek et al., 2022). Covalent triazine frameworks (CTFs) can be considered nitrogen-containing carbonaceous materials when derived from ionothermal synthesis where the ordered network of aromatic triazine rings represents only an idealized structure. Ionothermally-synthesized CTFs combine high porosity and surface area with good thermal and chemical stability, due to the strong covalent C-C and C-N bonds, as well as a high electrical conductivity of its extensively electronic structure. Therefore, CTFs are interesting carbon materials in electrocatalytic reactions, such as the HER and the ORR (Zhang et al., 2016; Tao et al., 2019; Zhang and Jin, 2019; Kamiya, 2020). The nitrogen content of CTFs enables them to act as catalysts by providing a high number of catalytic active sites, crucial for the performance of carbon electrocatalysts (Liu M. et al., 2019; Gunasekar and Yoon, 2020; Liu et al., 2020). Sönmez et al. tested several CTFs toward ORR with half-wave potentials starting at 680 mV (Sönmez et al., 2021). Jena et al. prepared a binaphthol-based CTF showing a half-wave potential of 737 mV toward ORR and an overpotential of 310 mV toward HER (Jena et al., 2020). Both studies can show that the electrocatalytic performance strongly depended on the quantity and configuration of nitrogen species acting as active sites. Synergistic effects between the nitrogen species are also discussed (Ning et al., 2019). In this regard, CTFs form a group of materials with adjustable properties (Tao et al., 2019). Doping with metals generates composites with improved electrocatalytic properties compared to bare materials. The strong nitrogen-metal interactions stabilize anchored M-NPs maintaining their high surface-to-volume ratio and allowing electronic interaction between the orbitals of the NP and the CTF support, promoting the overall activity of the electrocatalyst (Li and Antonietti, 2013; Yi et al., 2021). Siebels et al. stabilized rhodium and platinum NPs on a 1,4-dicyanobenzene-based CTF (CTF-1) and achieved overpotentials of 58 and 111 mV toward HER (Siebels et al., 2019). Öztürk et al. synthesized nickel NPs supported on CTF-1 demonstrating good ORR performances with half-wave potentials starting at 775 mV (Öztürk et al., 2020). Qiao et al. prepared molybdenum sulfide NP on CTF-1 achieving HER overpotentials starting at 93 mV (Qiao et al., 2019).

2,6-Dicyanopyridine-based CTFs (DCP-CTFs), provide an additional heteroatom with an unshared electron pair through the pyridine nitrogen atom, as a coordination site for M-NPs apart from the nitrogen atoms in the triazine ring. Zhang et al. tested different metal ions and clusters on DCP-CTF toward HER, with overpotentials starting at 46 mV for Pt containing DCP-CTF and 71 mV for Pd clusters (Zhang et al., 2021). Iwase et al. synthesized a copper-decorated DCP-CTF/Ketjen Black composite, showing a half-wave potential of 810 mV (Iwase et al., 2015). A similar approach was used by Zhou et al. with cobalt, reaching 830 mV (Zhou et al., 2021). To the best of our knowledge, the use of iridium or palladium compounds supported on CTFs toward HER and ORR is rarely researched. Although both metals are close in their electrocatalytic properties to platinum in terms of ORR and HER and provide comparable high performances (Shao et al., 2016; Sarkar and Peter, 2018; Chen et al., 2019; Zhu et al., 2020).

In this study, we present palladium and iridium oxide NPs supported on DCP-CTF, synthesized by microwave-assisted thermal decomposition reactions of metal precursors in the presence of the IL [BMIm][NTf_2_] and PC, and illustrate their high electrocatalytic activity toward HER and ORR.

### 1.1 Materials

2,6-Dicyanopyridine, DCP (2,6-pydridinedicarbonitrile, purity 97%) was obtained from Sigma Aldrich, anhydrous zinc chloride, ZnCl_2_ (98%) from Alfa Aesar, tetrairidium dodecacarbonyl, Ir_4_(CO)_12_ (98%) from Alfa Aesar and palladium acetylacetonate, Pd (acac)_2_ (99%) from abcr. Propylene carbonate (PC) (99.7%) was received from Carl Roth and dried under a high vacuum (<10^−6^ mbar) for 16 h before use. For the synthesis of 1-butyl-3-methylimidazolium bis(trifluoromethylsulfonyl)imide [BMIm][NTf_2_], 1-chlorobutane (>99%) from Alfa Aesar and N-methylimidazole (>99%) from Tokyo Chemical Industries were converted in a microwave reaction to [BMIm][Cl] (Aupoix et al., 2010). Lithium bis(trifluoromethanesulfonyl)imide, LiNTf_2_, from Fluorochem was subsequently used in an ion exchange reaction yielding the IL (Wasserscheid and Welton, 2007). Impurities were removed with active carbon and by washing with water. Afterward, the IL was dried under a high vacuum (<10^−6^ mbar) for 16 h. The purity was confirmed with ^1^H-/^13^C-NMR and anion chromatography to over 98%. The water content was determined twice by Karl-Fisher titration to less than 10 ppm for [BMIm][NTf_2_] and for PC, less than 1000 ppm. Graphitized carbon containing 20 wt% platinum (Pt^20^/C) was obtained from Sigma Aldrich for electrochemical performance comparison.

### 1.2 Methods

Powder X-ray diffractograms (PXRD) were measured on a Bruker D2–Phaser using a rotating flat silicon sample holder and Cu–Kα radiation (λ = 1.54182 Å, 30 kV). The averaged crystallite sizes ε were calculated by using the Scherrer equation (Eq. 1) with a Scherrer factor K=1 and selected reflexes at diffraction angles θ having half widths B . A variance was estimated from the differences in the crystallite sizes from different θ angles.

$$\varepsilon =\frac{K\times \lambda }{B\times \cos\left(\theta \right)}$$ (1)

^1^H- and ^13^C-NMR spectra were recorded on a Bruker Advance III-600 spectrometer using the residual proton solvent signal in acetonitrile-d_3_ versus TMS as reference (δ = 1.94 ppm in ^1^H, δ = 1.32 ppm in ^13^C NMR).

Ion chromatograms (IC) for [BMImNTf_2_] were collected with an ICS 1100 ion chromatograph equipped with an IonPac AS 22 analytical column (4 × 250 mm) and an AG 22 guard column (4 × 50 mm) from Dionex. The AERS 500 suppressor was regenerated with water. The injection volume was 25 μL and as eluent a mixture of 4.5 mmol L^−1^ sodium carbonate and 1.0 mmol L^−1^ sodium hydrogen carbonate with 30 vol% of acetonitrile has been used.

Coulometric Karl-Fischer titration for the water content in [BMImNTf_2_] and PC was conducted with an ECH AQUA 40.00 titrator from Analytik Jena. The measurements were performed with the headspace module, heated to 170°C, and sample containers sealed with crimp caps.

Nitrogen sorption experiments were carried out with a Nova 4000e from Quantachrome. The data were evaluated with the NovaWin 11.03 software. The Brunauer-Emmett-Teller (BET) surface areas were determined by selecting five adsorption points in the relative pressure range p/p_0_ of 0.05–0.3. The distribution of pore sizes was determined by non-local density functional theory (NLDFT) based on the "N_2_ at 77 K on carbon, slit pore (NLDFT) equilibrium model”. Theoretical (expected) surface areas S(BET)_calc._ For the NP@CTF composites were calculated by Eq. 2.

$$\text{S}{\left(\text{BET}\right)}_{\text{calc}\text{.}}=\frac{\text{wt\%}\;\text{of}\;\text{CTF}}{100\;}\text{×}\text{S}\left(\text{BET,CTF}\right)=\frac{100-\text{wt\%}\;\text{of}\;\text{M}}{100\;}\text{×}\text{S}\left(\text{BET,CTF}\right)$$ (2)

Thermogravimetric analysis (TGA) of the CTFs was performed with a Netzsch TG 209 F3 Tarsus operated from 30 to 1000 °C with synthetic air atmosphere, using a heating rate of 5 K min^−1^ and aluminum oxide crucibles as samples holders. Elemental (CNH) analyses of the CTFs were done with a Vario MICRO cube from Elementar Analysentechnik. Flame atomic absorption spectroscopy (AAS) for the Pd and Ir analysis was measured on a PinAAcle 900T from Perkin-Elmer. Samples for AAS were obtained by complete decomposition of defined amounts (∼3 mg) in 20 ml boiling aqua regia to which 20 ml of concentrated hydrochloric acid (HCl) was subsequently added. The reduced-volume solution was diluted with 20 ml semi-concentrated HCl, filtered, and transferred into a graduated flask. Ir samples were additionally treated with 2000 mg L^−1^ lanthanum using lanthanum (III) nitrate.

Scanning electron microscopy (SEM) images in combination with elemental mapping by energy-dispersive x-ray spectroscopy (SEM-EDX) were acquired on a JEOL JSM-6510 advanced electron microscope operating with a LaB_6_ cathode at 5–20 keV and Xflash 410 silicon drift detector from Bruker. Before measurements, samples were coated with gold (Au) by sputter deposition using a Jeol JFC 1200 fine coater.

Transmission electron microscopy (TEM) was conducted with an FEI Tecnai G2 F20 electron microscope operated at 200 kV accelerating voltage. TEM images were recorded with a Gatan UltraScan 1000P detector and TEM-EDX spectra with an EDAX detector system. TEM samples were prepared by diluting small amounts of material in acetonitrile and subsequently depositing the suspension on a 200 μm carbon-coated copper grid. The average size and size distribution from 300 individual particles was determined manually with the Gatan Digital Micrograph software. Diffraction images were calibrated with Debye–Scherrer patterns recorded from a gold reference sample (Luysberg et al., 2016).

X-ray photoelectron spectroscopy (XPS) was performed at a ULVAC-PHI VersaProbe II microfocus spectrometer equipped with an Al Kα X-ray source operating with 1486.8 eV. The C1s signal at 284.4 eV was taken as the reference for the binding energy scale. The evaluation of the spectra was done with the Casa XPS software, version 2.3.19PR1.0.

### 1.3 Synthesis of DCP-CTF600 and DCP-CTF750

The synthesis of covalent triazine frameworks from the monomer 2,6-pydridinedicarbonitrile was carried out as described in the literature (Artz et al., 2015; Tuci et al., 2017). Under inert (Ar) conditions anhydrous ZnCl_2_ (1.576 g, 11.6 mmol; 5 eq.) and 2,6-pydridinedicarbonitrile (0.300 g, 2.3 mmol, 1 eq.) were mixed and filled into a quartz glass ampoule. The ampoule was evacuated for at least 4 h, sealed, and thermally treated in a tube oven by different temperature programs. In the first step the material was heated for 10 h at 400°C (Tuci et al., 2017; Siebels et al., 2019; Öztürk et al., 2020). Subsequently, the ampoules were heated for 10 h at 600°C or 750°C. Afterward, the ampoule was opened and the black product was ground in a mortar and stirred in 100 ml millipore water for 5 days. The mixture was filtered and the collected black product was stirred in 2 mol L^−1^ HCl for 1 day. The washing procedure was continued with millipore water (3 × 75 ml), tetrahydrofuran (3 × 75 ml) and acetone (3 × 75 ml). The samples were dried under a high vacuum (<10^−6^ mbar) for 16 h, stored under an inert atmosphere (Ar) and designated according to the applied maximum temperature as DCP-CTF600 or DCP-CTF750.

### 1.4 Synthesis of Pd- and IrO_x_@CTF

Pd(acac)_2_ (28.6 mg, 94 µmol or 57.2 mg, 188 µmol) and 20 mg of DCP-CTF600/750 were dispersed in 2 g of [BMIm][NTf_2_] for 1 h in a microwave vial under inert conditions (Ar). Similarly, Ir_4_(CO)_12_ (14.4 mg, 52 µmol or 28.8 mg, 104 µmol) and 20 mg DCP-CTF were dispersed in 2 g of PC. The amount of the metal precursor was set to yield 33 or 50 wt% metal NPs in the composite. The mixtures were placed in a CEM Discover microwave and irradiated at 250°C with a power of 100 W for 20 min (Pd-NP) or 3 × 10 min (IrO_x_-NP) respectively. The resulting products were washed with 4 ml acetonitrile and centrifuged four times. Afterward, the dark products were dried under vacuum for 5 h. The samples were designated as Pd^XX^@CTF600/750^IL^ or IrO_x_^XX^@CTF600/750^PC^. The weight percentage (wt%) of metal in the composite was determined by AAS and is represented as superscript *XX* to the metal component.

### 1.5 Electrochemical measurements

Electrocatalytic measurements were conducted on an Interface 1010E potentiostat from Gamry Instruments. An RRDE-3A station from ALS Japan, a platinum counter electrode, and a glassy carbon (GC) electrode (5 mm diameter) were used for the three-electrode setup. The catalyst ink was prepared by dispersing 2.50 mg of the catalyst with 10 µl Nafion solution in 0.50 ml ethanol and subsequent sonication, resulting in a catalyst loading of 0.255 mg cm^−2^. For testing the samples toward HER, a nitrogen saturated 0.5 mol L^−1^ H_2_SO_4_ solution served as electrolyte and an Ag/AgCl reference electrode (stored in 3.5 mol L^−1^ KCl solution) was used. Potentials were related to the reversible hydrogen electrode (RHE) afterward. Measurements were performed at different potentials from 100 to -600 mV vs RHE with scan rates of 10 mV s^−1^ and, for stability tests, 100 mV s^−1^. Bubble formation at the GC surface was suppressed by using a rotation rate of 3600 rpm. The samples were activated with several cyclic voltammetry (CV) sweeps prior to the electrochemical measurements. Electrochemical impedance spectroscopy (EIS) was performed at −290 and −100 mV vs RHE in a frequency range from 1 to 100 kHz. For ORR experiments, a 1 mol L^−1^ O_2_-saturated potassium hydroxide electrolyte, a reversible hydrogen electrode from Gaskatel and a potential window from 1100 to 200 mV vs RHE was used instead. The rotation of the GC electrode was set to 1600 rpm. EIS was performed at 900 or 930 mV vs RHE in a frequency range from 1 to 100 kHz. All samples were measured twice to ensure reproducibility. Polarization curves were corrected by iR compensation.

## 2 Results and discussion

### 2.1 Characterization of DCP-CTF600 and DCP-CTF750

The monomer 2,6-dicyanopyridine (DCP) provides a comparatively high nitrogen content in the resulting CTF which is seen as crucial for good catalytic and NP stabilizing properties. We have synthesized DCP-CTF according to the literature by a fast ionothermal (ZnCl_2_) method using two different reaction temperatures (600 and 750°C; for further details see supporting information file (Kuhn et al., 2008; Artz et al., 2015; Tuci et al., 2017; Liu Y. et al., 2019).

The powder X-ray diffraction measurements of DCP-CTF600 and DCP-CTF750 illustrate the lack of crystallinity which is typical for CTFs synthesized by the ionothermal method at high temperatures due to the absence of a long-range order (Supplementary Figure SI1). The CHN elemental analysis yields the expected reduced nitrogen content which agrees with literature data (Supplementary Table SI1). Common to the ionothermal synthesis route, the obtained CTFs contain less nitrogen compared to the ideal structure due to partial decomposition with nitrogen loss (Kuhn et al., 2008; Siebels et al., 2019; Öztürk et al., 2020). Thermogravimetric analysis shows thermal stability of the CTFs up to 400°C. Typically, CTFs are synthesized at 400°C while at higher temperatures of typically 600°C the CTF materials become more essentially nitrogen-doped porous carbon materials (Öztürk et al., 2020). Noteworthy, the higher synthesis temperature leads to an increase in surface area, pore-volume, and pore size (Dey et al., 2017). High temperatures are also necessary to enhance the electrical conductivity of the CTF by graphitization (Liu M. et al., 2019; Öztürk et al., 2020). Nitrogen sorption measurements reveal a type I(b) isotherm for DCP-CTF600 and a combination of type I(b) to type II for the adsorption branch of DCP-CTF750 indicative of micro-porous materials and micro-to-macroporous materials with broader pore size distributions (Supplementary Figure SI2) including wider micropores (<2 nm) and narrow mesopores (2–50 nm), respectively (Thommes et al., 2015). The desorption branch of DCP-CTF750 has a small H4 loop which is often found for micro-mesoporous carbons. The BET surfaces area for DCP-CTF600 with 1334 m^2^ g^−1^ is significantly lower than for DCP-CTF750 with 2542 m^2^ g^−1^ in agreement with the literature (Supplementary Table SI2) (Artz et al., 2015; Dey et al., 2017; Tuci et al., 2017). Also, the total pore volume increased from 0.79 to 1.77 cm^3^ g^−1^ with the synthesis temperature. X-ray photoelectron spectroscopy (XPS) shows the formation of pyridinic, pyrrolic, oxidized, and quaternary/graphitic nitrogen (Supplementary Figure SI3). Our evaluation reveals that the amount of graphitic nitrogen with the binding energy at ∼ 400 eV and of oxidized nitrogen at ∼ 402 eV increases in particular with higher synthesis temperature. SEM pictures show the formation of shard-like particles with a layered structure having sizes in the micrometer range (Supplementary Figure SI4).

### 2.2 Characterization of Pd- and IrO_x_@CTF

**SCHEME 1:**
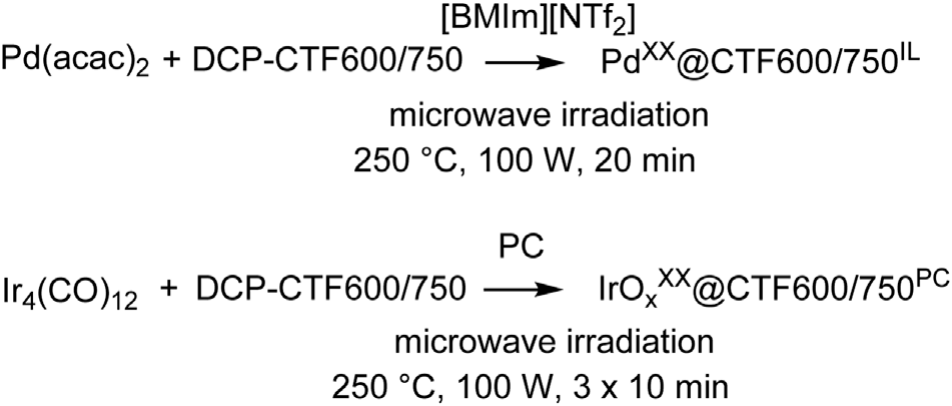
Microwave-assisted synthesis of Pd- and IrO_x_-NP supported on the dicyanopyridine (DCP) derived covalent triazine framework (CTF) in the ionic liquid (IL) [BMIm][NTf_2_] and in propylene carbonate (PC). The resulting weight percentage (wt%) of metal determined by AAS is represented as superscript *XX* to the metal component.

Microwave-assisted thermal decomposition reactions of metal complexes are established methods for the synthesis of metal NPs in ILs or PC (Vollmer et al., 2012; Janiak, 2013; Marquardt et al., 2014; Esteban et al., 2015a; Siebels et al., 2018). Besides the high absorption cross-section for microwave irradiation by ILs or PC also the formed metal NPs absorb microwave energy and become hot spots. This induces a rapid heating of the reaction mixture and a thereby high reaction rate (Bilecka and Niederberger, 2010; Vollmer et al., 2010). The Pd- and IrO_x_-NP supported covalent triazine frameworks in this work were synthesized by using Pd(acac)_2_ or Ir_4_(CO)_12_ as precursor materials for the formation of M-NPs (Scheme 1).

The CTF (DCP-CTF600 or DCP-CTF750) and the ratio of metal precursor to CTF were varied. The used amount of metal precursor loadings of 33 (in a 1:2 wt/wt approach) and 50 wt% (in a 1:1 wt/wt approach) could be theoretically achieved in the samples. The metal content in the samples was determined by flame AAS showing that a large amount of metal is deposited on the covalent triazine framework. Compared to the theoretical values, especially IrO_x_ samples show lower percentages of Ir, from 10—20 wt% only, indicating remaining metal particles or unreacted precursor in the dispersion. While Pd mass percent are almost in the theoretical range with 22—41 wt% (Table 1). With a higher reaction temperature during CTF synthesis, a higher metal loading was achieved in the subsequent microwave reactions. In the case of the Pd materials, the loading increased from 22 to 29 wt% and for the IrO_x_ materials from 10 to 14 wt%. Also, higher fractions of metal precursor in the reaction mixture, resulting in larger proportions of metal in the materials. Compared to a 1:2 metal-to-CTF ratio, the metal yields in a 1:1 approach are slightly lower in comparison to the theoretical values. In the following, the samples were designated according to their metal content.

**TABLE 1:** Metal content in the Pd- and IrO_x_@CTF materials.

Material^a^	Solvent	Theor. Metal content (wt%)^b^	Metal content determined by AAS (wt%)	Yield of metal deposit (%)^c^
Pd^22^@CTF600^IL^	[BMIm][NTf_2_]	33	22	67
Pd^29^@CTF750^IL^	[BMIm][NTf_2_]	33	29	88
Pd^41^@CTF750^IL^	[BMIm][NTf_2_]	50	41	82
IrO_x_^10^@CTF600^PC^	PC	33	10	30
IrO_x_^14^@CTF750^PC^	PC	33	14	42
IrO_x_^20^@CTF750 ^PC^	PC	50	20	40

a The weight percentage (wt%) of metal determined by AAS is represented as superscript *XX* to the metal component.

b Possible metal content for quantitative metal precursor decomposition and deposition.

c AAS-determined metal content divided by theor. metal content.

**FIGURE 1:**
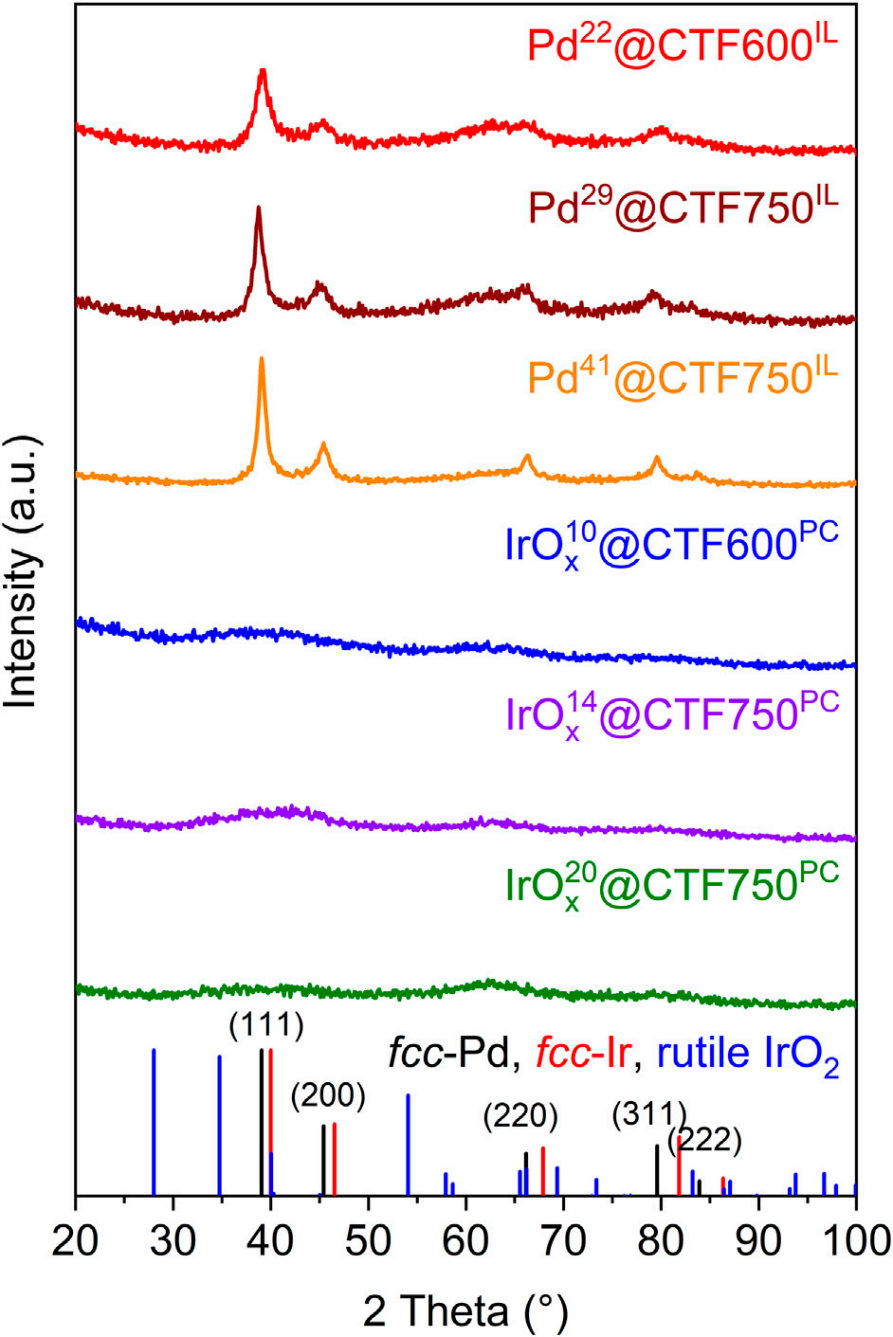
PXRD patterns of Pd@CTF and amorphous IrO_x_@CTF materials synthesized in [BMIM][NTf_2_] or PC. The reference diffractograms of fcc-Pd, fcc-Ir, and rutile IrO_2_ were adapted from the crystallographic open database cif-files (CrOD 1011104 for Pd, CrOD 1534947 for Ir and CrOD 1538153 for IrO_2_).

Powder X-ray diffraction (PXRD) measurements confirm the reproducible formation of face-centered cubic Pd showing broad reflexes which indicate small crystallites (Figure 1). For crystallite size calculations the Scherrer equation was used with reflexes that correspond to the (111) (200), (220), and (311) planes (Eq. 1). The crystallite sizes from each reflex were subsequently averaged and a variance was determined. The calculated crystallite size remained essentially invariant from 4 ± 2 to 5 ± 2 nm when going from CTF600 to CTF750 with Pd^22^ to Pd^29^. This indicates that the presence of larger pore sizes in CTF750 does not necessarily lead to a bigger crystallite size. However, a higher amount of metal precursor leads to an increased crystallite size of 8 ± 2 nm, when going from the Pd^29^ to Pd^41^, respectively. A reason for this could be a declining stabilization effect of the IL due to the higher amount of metal in the dispersion and an NP saturated CTF surface. In contrast, IrO_x_ samples show no reflexes, indicating amorphous particles (Pfeifer et al., 2016; Jiang et al., 2019).

**FIGURE 2:**
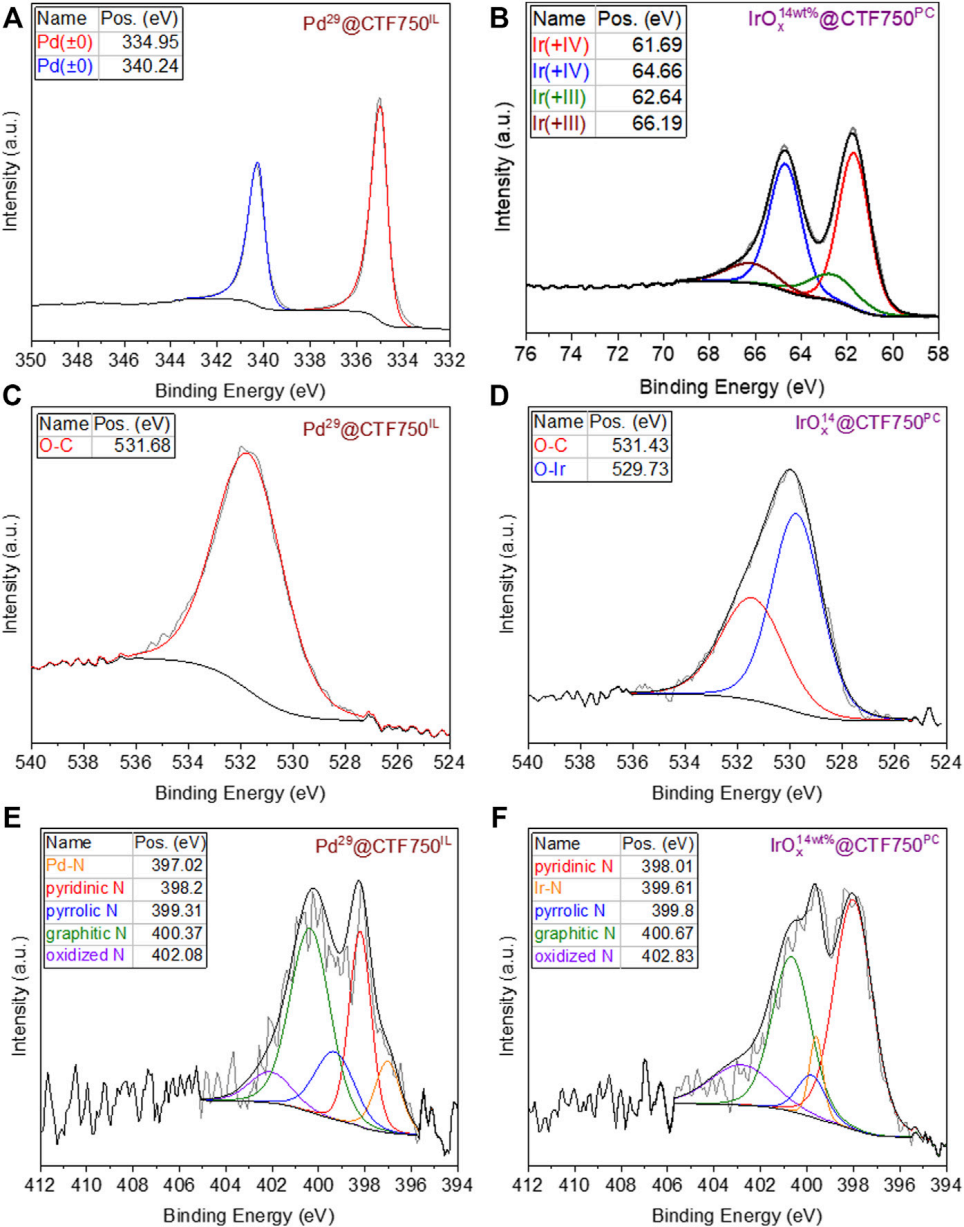
High resolution X-ray photoelectron spectra before electrocatalysis of Pd- and IrO_x_-NPs supported DCP-CTF, showing **(A)** Pd 3d and **(B)** Ir 4f orbitals and the corresponding spectra of **(C,D)** the O 1s orbital and **(E,F)** the N 1s orbital.

The chemical composition and state of selected Pd@CTF and IrO_x_@CTF materials were further investigated by XPS (Figure 2). For Pd^29^@CTF750^IL^ the zero-valent oxidation state of palladium metal from the reductive decomposition of Pd^II^(acac)_2_ could be proven with the high-resolution spectra for Pd 3d with binding energies of 334.95 and 340.24 eV (doublet separation of 5.29 eV) (Figure 2A) (Moulder et al., 1992; Yang et al., 2014). While for IrO_x_^14^@CTF750^PC^ two different oxidation states were confirmed from the deconvolution of the high-resolution spectrum (Figure 2B). The binding energies at 61.69 and 64.66 eV (doublet separation of 2.97 eV) are in good agreement with the literature for the 4f_7/2_ state of Ir (+IV) and Ir (+III) which suggests a mixed valent oxide (Hall and Sherwood, 1984; Pfeifer et al., 2016; Jürgensen et al., 2020; Park et al., 2020). According to the peak fitting, the Ir(+IV) state is present with 76 at%, and the Ir(+III) state with 24 at%. Furthermore, the high-resolution spectrum for IrO_x_^14^@CT750^PC^ reveals the absence of metallic iridium or remaining iridium (0) precursor for which the binding energies would be expected at 60.9 eV (doublet separation of 2.98 eV) (Yang et al., 2014). The O 1s high-resolution spectrum of Pd^29^@CTF750^IL^ demonstrates carbon-bound oxygen belonging to the CTF at a binding energy of 531.68 eV (Figure 2C) while for IrO_x_^14^@CTF750^PC^ additional metal-bound oxygen is detected at a lower binding energy of 529.73 eV (Figure 2D) (Jürgensen et al., 2020). Notably, the oxidation of bulk iridium metal is expected at 400 °C under atmospheric air, therefore oxidation during handling in air can be excluded (Zhu et al., 2014). However, XPS analysis shows that the iridium (0) precursor is oxidized when synthesized in oxygen-containing propylene carbonate, PC. This can be attributed to the formation of nanoparticular structures having a high reducing reactivity toward the carbonate group in PC such that the presumably initially formed nano-iridium(0) is oxidized. The peak fitting of the N 1s high-resolution spectrum (Figure 2E) shows an additional peak at 397.02 eV in the Pd sample associated with the Pd-N interaction of the Pd-NP and the CTF support (Chen et al., 2020). The fitted spectrum of the Ir sample (Figure 2F) shows a peak at 399.61 eV which can be attributed to the Ir-N interaction (Li and Antonietti, 2013). Moreover, the survey spectra also exhibit the expected signals of carbon and nitrogen belonging to the CTF (Supplementary Figure SI7). Only for Pd^29^@CTF750^IL^ a small residue of the ionic liquid can be concluded from the fluorine signal.

**FIGURE 3:**
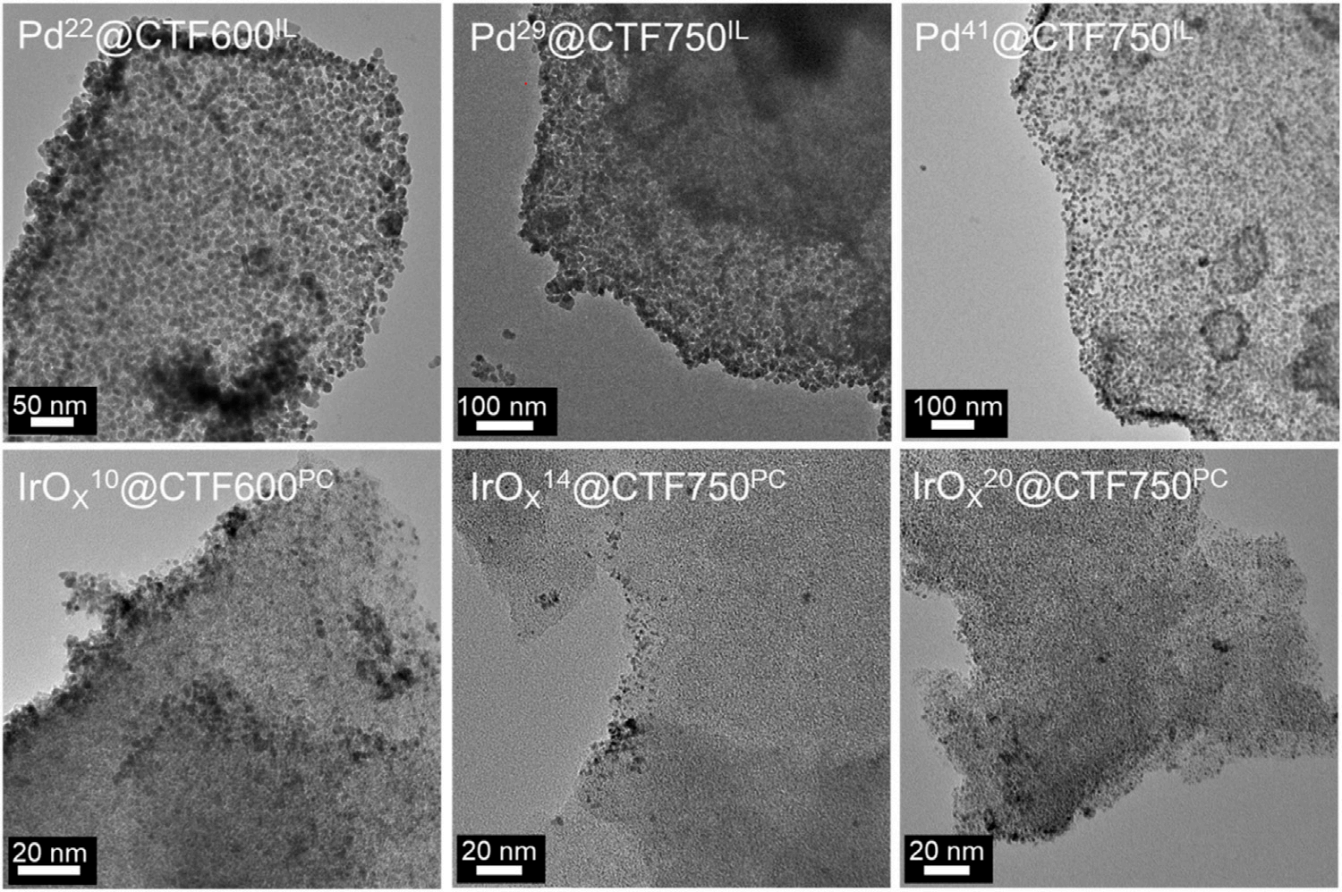
TEM images of Pd- and IrO_x_-NP supported DCP-CTF materials before electrocatalysis.

Transmission electron microscopy (TEM) was performed for the characterization of the synthesized samples toward morphology and size of the nanoparticles. TEM images in Figure 3 show the distribution of nanoparticles on CTF flakes, with the latter having a lower contrast compared to the M-NPs. The sizes of 300 particles were measured and subsequently evaluated. For Pd-containing samples, average particle sizes with size dispersions of 11 ± 2 to 13 ± 2 nm were determined, in good agreement with the respective crystallite sizes from PXRD and the Scherrer equation (Table 1). The crystallite size is expected to be smaller than the particle size from TEM since several crystallites can form a particle. Among the Pd samples, the average crystallite and particle size increases with the Pd content while the presence of larger pores in DCP-CTF750 compared to DCP-CTF600 does not affect the particle size. The IrO_x_ samples show similar average particle sizes in the range of 2 ± 1 nm obviously also unaffected by metal loading and the pore size of the CTF. For both Pd and IrO_x_ the particle size distributions can be considered narrow for syntheses of nanoparticles in IL or PC (Supplementary Figure SI8) (Schütte et al., 2014; Esteban et al., 2015b; Schütte et al., 2017; Siebels et al., 2018). Furthermore, the TEM images show exfoliated CTF sheets. The exfoliation of carbon-type materials emerges during microwave heating in IL and PC (Marquardt et al., 2011; Marquardt et al., 2014; Esteban et al., 2015a). The exfoliation improves the accessibility of the *in situ* formed NPs to the CTF surface (Wang et al., 2010; Siebels et al., 2019). Energy-dispersive X-ray spectroscopy (EDX) measurements confirm the high purity of the samples showing here no remaining zinc chloride and only negligible amounts of IL in the NP@CTF samples (Supplementary Figure SI9). Selected area electron diffraction (SAED) is presented as an example for Pd^29^@CTF750^IL^ showing ring patterns of a polycrystalline sample belonging to the (111) (200), (220) and (311) planes of Pd metal (Supplementary Figure SI9). In the case of the amorphous IrO_x_-containing samples, SAED did not show diffraction patterns.

**FIGURE 4:**
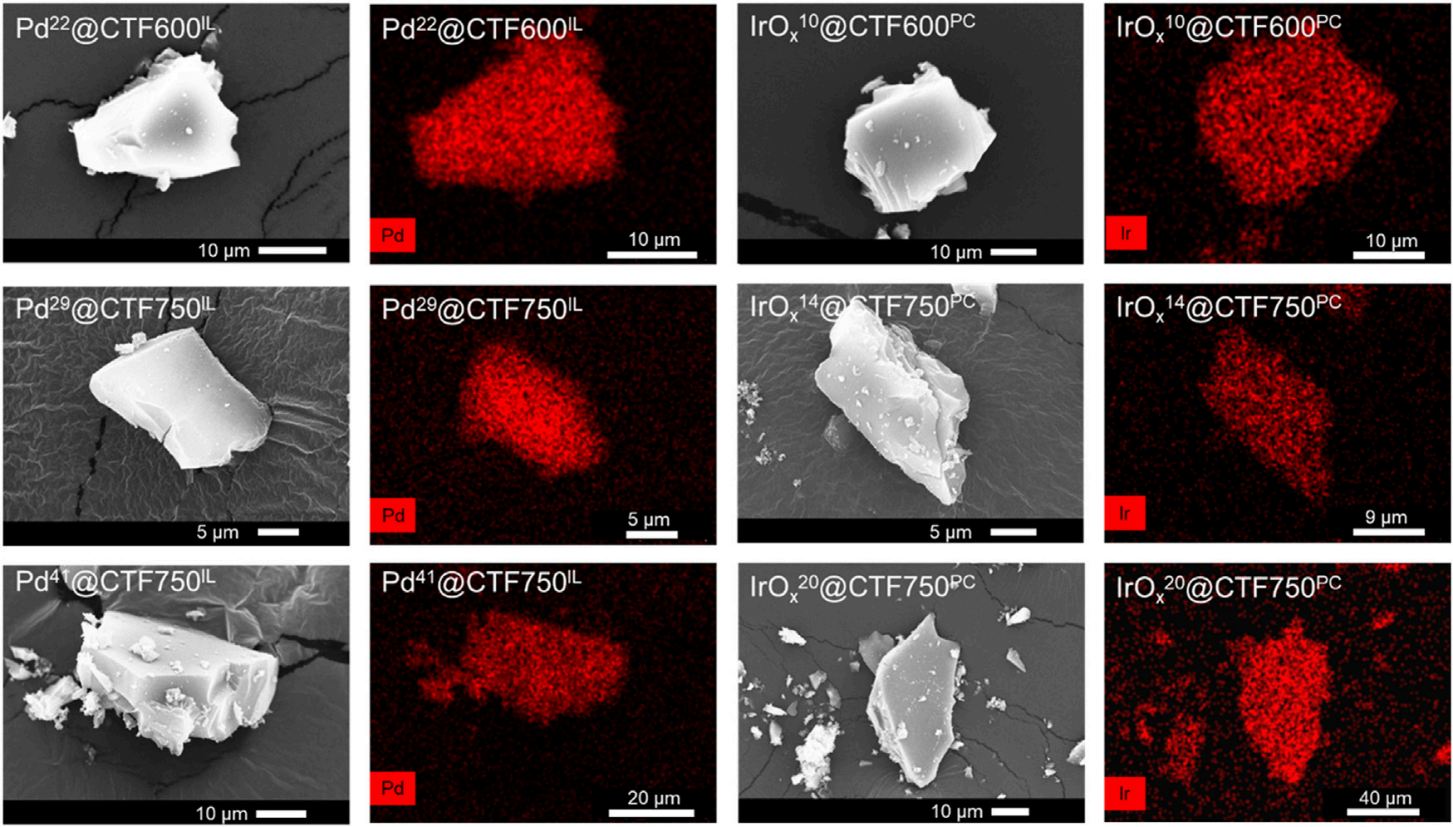
SEM images and EDX Pd and Ir mappings (in red) for Pd- and IrO_x_@CTF composites.

Further characterization regarding morphology was done by scanning electron microscopy (SEM) and SEM-EDX mapping. Pictures of the samples show a uniform and complete coverage of Pd or IrO_x_ on the surface of shard-like CTF particles (Figure 4). Similar to the TEM results, no minor amounts of metal could be detected by SEM-EDX outside the CTF particles, which also demonstrates the good stabilization properties of DCP-CTF600 and DCP-CTF750.

**FIGURE 5:**
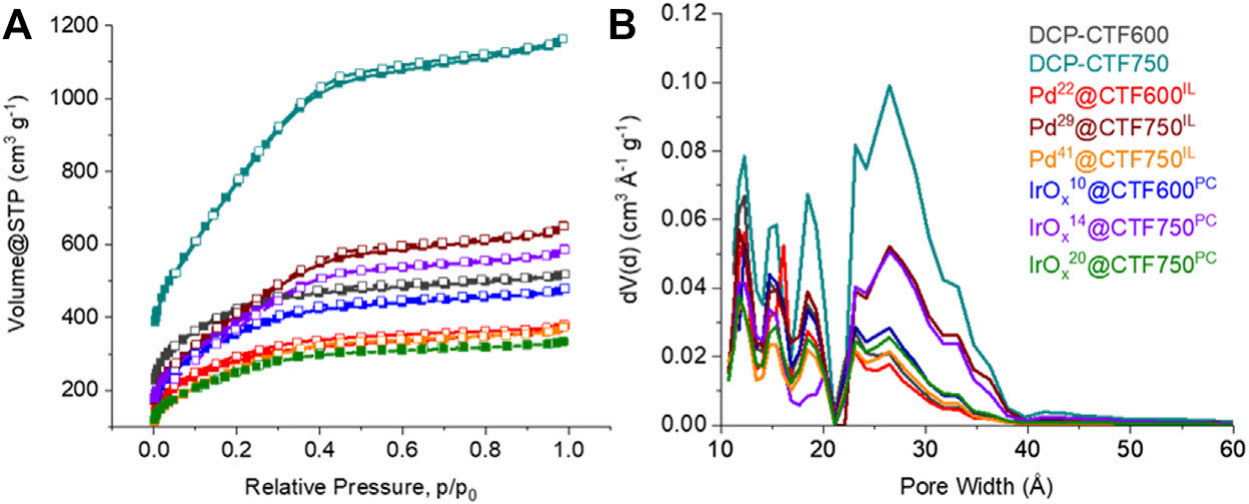
**(A)** Nitrogen adsorption (filled symbols) and desorption isotherms (empty symbols) and **(B)** pore size distribution curves of the Pd- and IrO_x_-NP decorated DCP-CTF materials and native CTFs.

The porosity of the CTF and Pd- and IrO_x_@CTF materials was investigated by nitrogen sorption measurements and yielded a type I(b) isotherm, typical for microporous materials (<2 nm), with contribution from type II at higher relative pressure for mesopores (2—50 nm) (Figure 5) (Thommes et al., 2015). The isotherm shapes of Pd- or IrO_x_@CTF resemble the shapes of the neat CTF isotherms, with expected lower uptakes. Consequently, the porosity in the metal-loaded materials decreased compared to the native CTFs. While DCP-CTF600 and -750 achieved BET surface areas of 1334 and 2542 m^2^ g^−1^ and pore volumes of 0.79 and 1.77 cm^3^g^–1^, respectively, the NP-supported materials have surface areas of 904–1353 m^2^ g^−1^ and pore volumes from 0.50 to 0.98 cm^3^ g^−1^, due to the nonporous metal content (Table 1). A high surface area and a high pore volume are essential requirements for mass transport and ion conductivity in electrocatalysis (Zeng and Li, 2015). Furthermore, the BET surface decreases with a higher metal loading, that is in the case of Pd^29^@CTF750^IL^ and Pd^41^@CTF750^IL^ from 1353 to 971 m^2^ g^−1^, and for IrO_x_^14^@CTF750^PC^ and IrO_x_^20^@CTF750^PC^ from 1229 to 918 m^2^ g^−1^. It should be mentioned that the expected BET surface areas and pore volumes based on the CTF content are higher than the experimental values (Eq. 2, Supplementary Table SI3). The lower-than-expected porosity of the samples can be attributed to pore-blocking caused by the NPs. Pore blocking by remaining IL can be excluded since PC which should be fully removable through evacuation gives the same lower than expected porosity. Also, the exfoliation of the CTF layers in IL and PC reduces the porosity of the CTFs but provides the increased outer surface area for the deposition of NPs (Siebels et al., 2019).

### 2.3 Hydrogen evolution reaction

**FIGURE 6:**
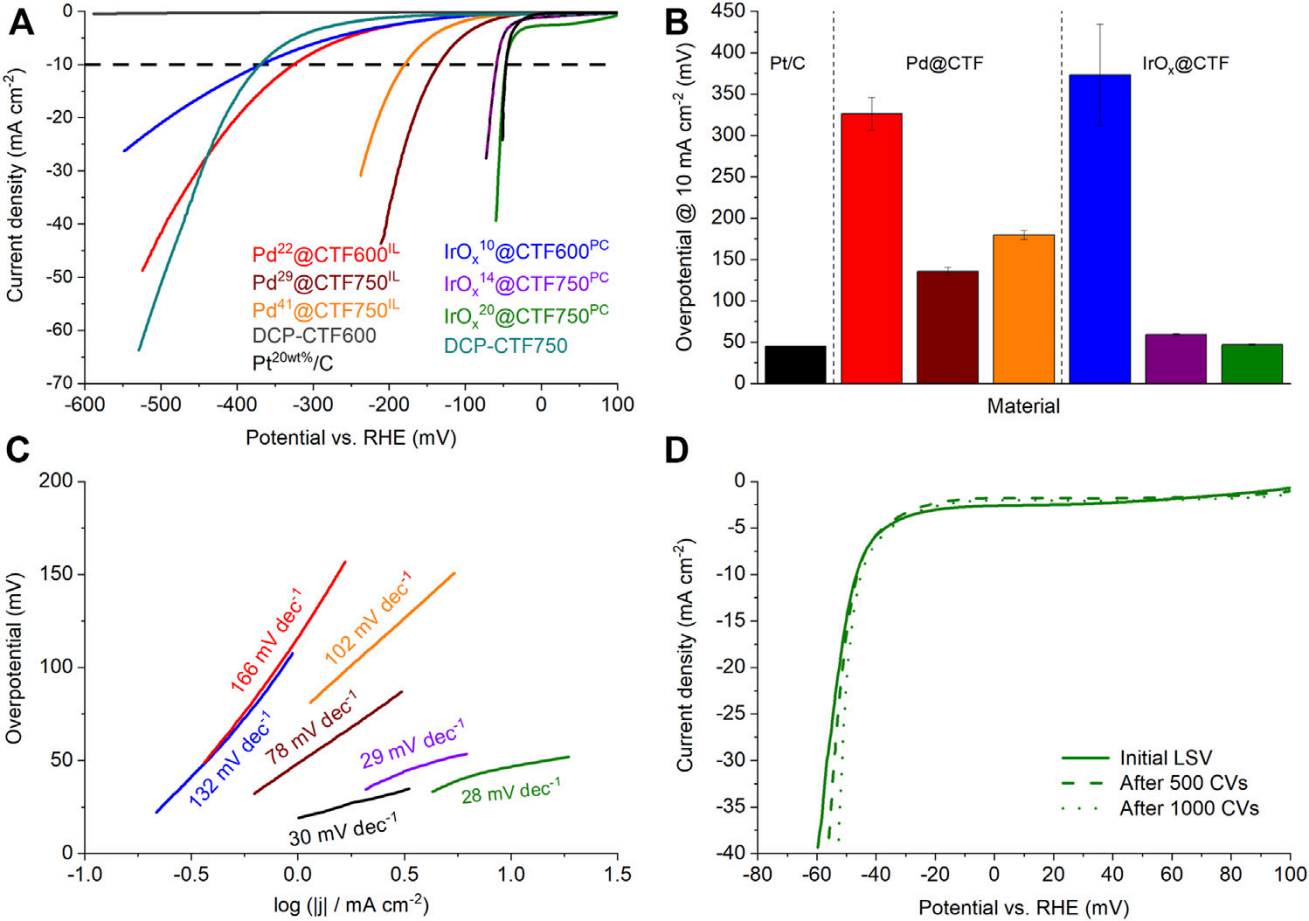
**(A)** HER polarization curves, **(B)** overpotentials determined at 10 mA cm^−2^, and **(C)** Tafel plots of the Pd- and IrO_X_@CTF materials in 0.5 mol/L H_2_SO_4_. **(D)** Polarization curves of IrO_x_^20^@CTF750^PC^ after CV stability test.

To investigate the electrocatalytic activity of the Pd- and IrO_x_@CTF samples toward HER, electrochemical measurements were performed in a 0.5 mol L^−1^ H_2_SO_4_ electrolyte and the obtained results compared with commercial Pt^20^/C (Siebels et al., 2019). The latter can be regarded as a benchmark material in electrocatalysis for HER and ORR (Zeng and Li, 2015; Kocha et al., 2017; Woitassek et al., 2022). Figure 6A presents the polarization curves of the samples after activation. Here, in particular, IrO_x_^14^@CTF750^PC^ and IrO_x_^20^@CTF750^PC^ demonstrate curves which are very close to the theoretical starting point of the HER at 0 V vs RHE followed by Pd^29^@CTF750^IL^ and Pd^41^@CTF750^IL^. Furthermore, results show low performances in the selected potential area for native DCP-CTF600 samples. Until –600 mV neat (metal-free) DCP-CTF600 achieved only a small current density of 0.45 mA cm^−2^. Thus, the overpotential at 10 mA cm^−2^, $\eta_{10\;mA\;cm^{–2}}^{HER}$ , could not be measured in the selected potential area. Also, DCB-CTF750 alone shows a high overpotential of 370 mV to achieve 10 mA cm^−2^ (Figure 6B). The difference between CTF600 and CTF750 can be explained by the increased graphitization of the CTF at 750°C, which leads to a higher electrical conductivity (Öztürk et al., 2020). Also, the higher surface area and structural defects caused by the increased temperature during ionothermal synthesis give rise to better electrocatalytic performance. Several studies show that the nitrogen species notably affect the electrocatalytic performance of CTF-based materials. Especially quaternary/graphitic nitrogen is discussed as performance-boosting species (Jena et al., 2020; Sönmez et al., 2021). Compared to native CTF600, the materials Pd^22^@CTF600^IL^ and IrO_x_^10^@CTF600^PC^ have already significantly lower overpotentials with 325 and 368 mV at 10 mA cm^−2^, respectively. Accordingly, the metal load improves the electrocatalytic properties of DCP-CTF600 significantly, with Pd^22^@CTF600^IL^ being better than IrO_x_^10^@CTF600^PC^.

IrO_x_^14^@CTF750^PC^ and IrO_x_^20^@CTF750^PC^ show very low overpotentials with 59 and 47 mV at 10 mA cm^−2^, respectively, and compete with the performance of Pt^20^/C having an overpotential of 46 mV. Thereby, the overpotential of Pt^20^/C is in good accordance with the literature (Qiao et al., 2019; Siebels et al., 2019). These results present an outstanding performance of IrO_x_-NP decorated CTFs as electrocatalysts toward HER (Supplementary Table SI4). The observed overpotentials of the Pd samples are 135 mV for Pd^29^@CTF750^IL^ and 180 mV for Pd^41^@CTF750^IL^ indicating a tradeoff relation between the amount Pd and the electrocatalytic performance. This can be explained by blocked pores, reduced active sites, or an increased amount of M-NP not deposited on the conductive CTF (since the overall mass of M-NP@CTF catalyst in the ink is same) causing reduced electrocatalytic performance. A similar tradeoff phenomenon was observed in the study of Qiao et al. (Qiao et al., 2019).

Besides low overpotentials, a good electrocatalyst should have low Tafel slopes, which characterize the sensitivity of the current to an applied potential (Zeng and Li, 2015). The Tafel slope also allows insights into the rate-determining reaction path. Figure 6C shows the Tafel plots of the samples based on the kinetically controlled areas at low overpotentials. Again, the samples are led by the superior performance of IrO_x_^20^@CTF750^PC^ with 28 mV dec^−1^ indicating very fast kinetics and the Tafel reaction 2 M-H* ⇆ 2M + H_2_ as a rate-determining step (∼30 mV dec^−1^). This sample is followed by IrO_x_^14^@CTF750^PC^ with 29 mV dec^−1^. Pt^20^/C with 30 mV dec^−1^. Pd^29^@CTF750^IL^ with 78 mV dec^−1^ shows slower kinetics which can be assigned to the Heyrovsky reaction M-H* + e^–^ + H^+^ ⇆ M + H_2_ (∼40 mV dec^−1^) as a rate-determining step. The slopes of the other samples partially exceed 120 mV dec^−1^ indicating the Volmer process M + e^–^ + H^+^ ⇆ M-H* as a rate-determining step and a low sensitivity to the applied potential (Table 2).

**TABLE 2:** Average crystallite and particle sizes and porosity of Pd- and IrO_x_@CTF materials.

Material	Crystallite size (nm)^a^	NP size (nm)^c^	BET surface area (m^2^ g^−1^)^d^	Total pore volume (cm^3^ g^−1^)^e^
DCP-CTF600	−	−	1334	0.79
DCP-CTF750	−	−	2542	1.77
Pd^22^@CTF600^IL^	4 ± 2	11 ± 2	904	0.56
Pd^29^@CTF750^IL^	5 ± 2	11 ± 2	1353	0.98
Pd^41^@CTF750^IL^	8 ± 2	13 ± 3	971	0.55
IrO_x_^10^@CTF600^PC^	^b^	2 ± 1	1055	0.72
IrO_x_^14^@CTF750^PC^	^b^	2 ± 1	1229	0.88
IrO_x_^20^@CTF750 ^PC^	^b^	2 ± 1	918	0.50

a Determined from PXRD and calculated by the Scherrer equation with Scherrer factor = 1 using the Pd-metal reflexes at (111), (200), (220) and (311).

b No reflexes present for amorphous IrO_x_.

c Determined by TEM. The mean size and size distribution (Fig. SI8) were manually measured from 300 particles.

d From N2 sorption at 77 K, cf. Fig. 5a. For BET, calculation five adsorption points between p/p0 = 0.05–0.3 were selected.

e Determined at p/p0 = 0.95.

To check the longer-term stability in HER, a cyclic voltammetry (CV) durability test comprising 1000 cycles was carried out exemplary on IrO_x_^20^@CTF750^PC^ which demonstrated the best performance among the NP@CTF samples regarding HER (Figure 6D). Even after 1000 CV cycles the sample showed a stable overpotential with 45 mV at 10 mA cm^−2^ exhibiting superior stability. TEM investigations after these 1000 cycles demonstrate no significant change in the average particle size of the IrO_x_-NPs with 2 ± 1 nm (Supplementary Figure SI10) and thus show good stabilization by the metal-nitrogen interaction.

Electrochemical impedance spectroscopy (EIS) at defined potentials was performed before the stability test. Data were plotted in a Nyquist plot and fitted to a Voigt circuit model with several circuit elements (Supplementary Figure SI12). While Pd^22^@CTF600^IL^ and IrO_x_^10^@CTF600^PC^ show high charge-transfer resistances, R_ct_, with 38.4 and 55.8 Ω, respectively, Pd29@CTF750IL with 4.6 Ω and Pd^41^@CTF750^IL^ with 6.4 Ω have lower charge transfer resistances. IrO_x_^14^@CTF750^PC^ and IrO_x_^20^@CTF750^PC^ demonstrate the lowest resistances with 4.1 and 2.3 Ω, respectively (Supplementary Table SI4).

### 2.4 Oxygen reduction reaction

**FIGURE 7:**
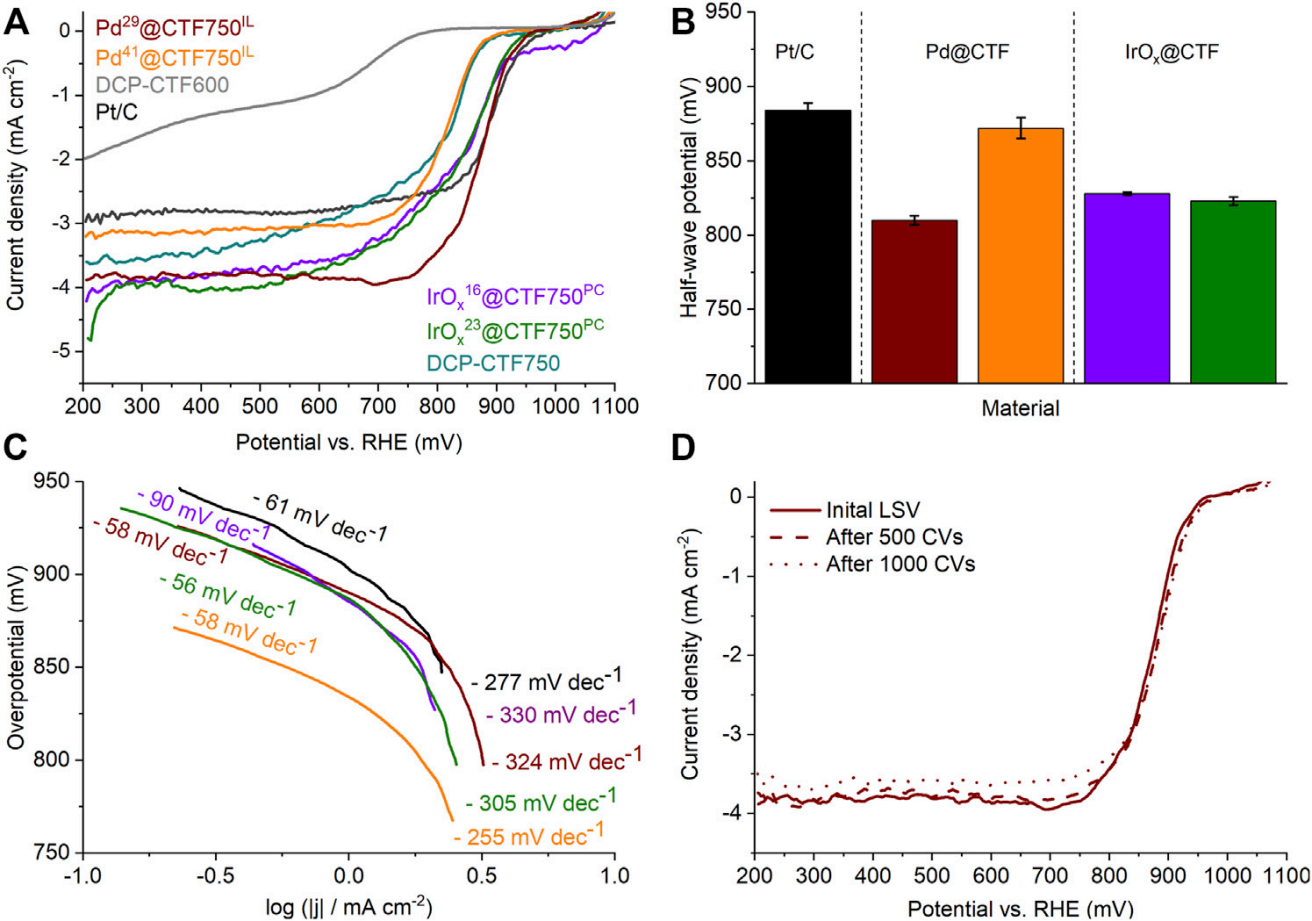
**(A)** ORR polarization curves, **(B)** half-wave potentials, and **(C)** Tafel plots of the Pd- and IrO_X_@CTF samples in 1.0 mol/L KOH. **(D)** Polarization curves of Pd^29^@CTF750^IL^ after CV stability test.

The ORR was only investigated for the DCP-CTF750 series because of their generally better performance over the DCP-CTF600 materials (Figure 7A). Measurements were performed twice in O_2_-saturated 1.0 mol L^−1^ KOH electrolyte and compared with commercial Pt^20^/C (Liang et al., 2012). Figure 7A shows the polarization curves of the tested samples after activation with the typical kinetic, mixed-kinetic-diffusion, and diffusion-controlled regions (Xia et al., 2016). Here, the Pd- and IrO_x_@CTF750 materials demonstrate outstanding current densities compared to Pt^20^/C. As shown in Figure 7A, the current densities at 400 mV, $i_{400\;mV}^{ORR}$ , especially for IrO_x_^14^@CTF750^PC^ with 3.8 mA cm^−2^ and IrO_x_^20^@CTF750^PC^ with 4.1 mA cm^−2^ are very high and are followed by Pd^29^@CTF750^IL^ with 3.8 mA cm^−2^and Pd^41^@CTF750^IL^ with 3.1 mA cm^−2^. The high currents especially for the IrO_x_-containing samples can be attributed to the small particle sizes. For Pd samples, the high metal content in particular influences the measured current density similar to the results of the HER measurements.

The half-wave potential, $E_{1/2}^{ORR}$ , of Pd^29^@CTF750^IL^ is more positively shifted within the prepared materials, indicating a better ORR activity (Figure 7B) (Öztürk et al., 2020). As for the IrO_x_-containing materials, a higher metal content does not directly relate to an increased ORR activity in terms of the measured current densities and half-wave potentials. Noteworthy, the current density and the half-wave potential do not increase significantly with increased metal content in the IrO_x_@CTF750 series. In contrast, Pd^41^@CTF750^IL^ has a much lower half-wave potential with 810 mV in comparison to Pd^29^@CTF750^IL^ with 872 mV (Table 3). This could be related to the blocking of active sites by the high metal loading similar to the HER results. According to these conclusions from ORR and HER measurements, we assume that the synergistic effect of M-NP and CTF is characterized by an optimum in the loading or relative ratio. Furthermore, the comparison of the measured half-wave potentials for the different metal species results in a similar trend as in the literature for Pt, Pd, and Ir (Rahman et al., 2021). For Pt^20^/C a current density of 2.8 mA cm^−2^ and a half-wave potential of 884 mV could be measured in the 1.0 mol L^−1^ KOH electrolyte, which is comparable to the literature (Yan et al., 2015; Öztürk et al., 2020). Note that the current density is strongly influenced by the concentration of the electrolyte. A high KOH concentration is associated with a reduced O_2_ solubility and diffusion resulting in a reduced current density. Furthermore, especially for Pt/C the formation of PtOH is favored at higher electrolyte concentrations (Jin et al., 2010; Yan et al., 2015; Iwai et al., 2019). Here, the synthesized materials demonstrate competitive performance (Supplementary Table SI5).

**TABLE 3:** HER and ORR parameters of the Pd- and IrO_x_@CTF materials.

Material^a^	$\eta_{10\;mA\;cm^{–2}}^{HER}\;$ (mV)^b^	Tafel slope (mV dec^−1^)	$E_{1/2}^{ORR}$ (mV)^c^	$i_{400mV}^{ORR}$ (mA cm^−2^)^d^	Tafel slope (mV dec^−1^)
DCP-CTF600	>600	−	663	1.3	−
DCP-CTF750	370	162	800	3.4	58/339
Pd^22^@CTF600^IL^	325	166	−	-	−
Pd^29^@CTF750^IL^	135	78	872	3.8	58/324
Pd^41^@CTF750^IL^	180	102	810	3.1	58/313
IrO_x_^10^@CTF600^PC^	368	132	−	−	−
IrO_x_^14^@CTF750^PC^	59	29	823	3.8	90/330
IrO_x_^20^@CTF750^PC^	47	28	828	4.1	56/305
Pt^20^/C	46	30	884	2.8	61/277

a HER was conducted in N_2_ saturated 0.5 mol L^−1^ H_2_SO_4_ and ORR in O_2_ saturated 1.0 mol L^−1^ KOH with potentials related to the RHE. Outstanding values are highlighted in bold.

b Overpotential in HER at 10 mA cm^−2^.

c Half-wave potential in ORR.

d Current density in ORR at 400 mV.

Tafel plots for ORR with NP@CTF750 are presented in Figure 7C and are characterized by a lower and a higher potential region. Similar to HER, smaller Tafel slopes for ORR indicate fast kinetics and a better electrocatalytic activity (Siow et al., 2021). Here, IrO_x_^20^@CTF750^PC^ demonstrates an outstandingly low Tafel slope with 56 mV dec^−1^ in the lower overpotential region and 305 mV dec^−1^ at higher potentials. Pt^20^/C shows comparable results to the literature with 61 and 277 mV dec^−1^ for the lower and higher potential region (Park et al., 1986; Shinagawa et al., 2015).

Stability toward ORR was tested by a cyclic voltammetry (CV) durability test comprising 1000 cycles and was exemplary carried out on Pd^29^@CTF750^IL^ (Figure 7D). After 1000 CV cycles the sample shows a nearly unchanged half-wave potential with 879 mV and a current density of 3.6 mA cm^−2^, demonstrating excellent stability in 1 mol L^−1^ KOH electrolyte. TEM measurements after electrocatalysis yield a slight increase in the average particle size from 11 ± 2 to 14 ± 3 nm which may indicate an agglomeration of particles during electrocatalysis (Supplementary Figure SI10).

The data from electrochemical impedance spectroscopy (EIS) (Supplementary Figure SI12) was plotted in a Nyquist plot and fitted to a simplified Voigt circuit model to obtain a quantitative date for the charge transfer resistance (Ruiz-Camacho et al., 2017). Based on this model for Pd^29^@CTF750^IL^ a low charge transfer resistance of 284 Ω at 900 mV vs RHE was obtained (Table 4).

## 3 Conclusion

Pd and IrO_x_ nanoparticles deposited on a nitrogen-rich CTF were prepared by a simple and energy-saving microwave reaction route using the IL [BMIm][NTf_2_] or propylene carbonate as reusable solvents without further additives. Pd(acac)_2_ and Ir_4_(CO)_12_ served as metal precursors. The CTF supports were synthesized by the fast ionothermal (ZnCl_2_) method at 600 and 750 °C Deposition of Pd- and IrO_x_-NPs on exfoliated CTF layers was proven by PXRD, XPS, SEM, and TEM measurements.

The materials demonstrate competitive electrocatalytic activities toward HER and ORR compared to commercial Pt^20^/C. Here, the amount of metal NP and the characteristics of the CTF influence the performance during electrocatalysis. For HER a higher temperature during CTF synthesis results in a smaller overpotential due to partial graphitization. Moreover, a higher metal loading increases the overpotential due to a lower porosity, blocked active sites, and larger nanoparticles. In particular, IrO_x_ samples could achieve very low overpotentials of 47–368 mV and Tafel slopes of 28–132 mV dec^−1^, matching the performance of Pt^20^/C. After a stability test with 1000 CVs, the good performance of IrO_x_^20^@CTF750^PC^ persists. In comparison, Pd samples demonstrate higher overpotentials from 135 to 325 mV and Tafel slopes from 78 to 166 mV dec^−1^. As expected for ORR, the best performance can be assigned to Pd^29^@CTF750^IL^ with a half-wave potential of 872 mV and a good stability after 1000 CVs. Here, IrO_x_-samples show higher current densities up to 4.1 mA cm^−1^ at 400 mV and half-wave potentials of 823 and 827 mV. Striking is the low performance of Pd^41^@CTF750^IL^ with a half-wave potential of 810 mV which can be attributed to reduced active sites and larger NP sizes.

This study shows that the preparation of M-NP decorated CTFs by microwave reactions is a promising opportunity to efficiently synthesize materials with high electrocatalytic activity and stability.

## Data Availability

The raw data supporting the conclusion of this article will be made available by the authors, without undue reservation.
