# Supplementary material for: Microwave-assisted synthesis of iridium oxide and palladium nanoparticles supported on a nitrogen-rich covalent triazine framework as superior electrocatalysts for the hydrogen evolution and oxygen reduction reaction
Source: Front Chem. 2022 Jul 26;10:945261. doi: 10.3389/fchem.2022.945261 (PMC9360555; doi:10.3389/fchem.2022.945261)
Supplement: Supplementary file 1 [file DataSheet1.docx]

Supplementary Material

**Microwave-assisted synthesis of iridium oxide and palladium nanoparticles supported on a nitrogen-rich covalent triazine framework as superior electrocatalysts for the hydrogen evolution and oxygen reduction reaction**

Lars Rademacher,^a^ Thi Hai Yen Beglau,^a^ Tobias Heinen,^a^ Juri Barthel,^b^ and Christoph Janiak*^a^

^a^Institut für Anorganische Chemie und Strukturchemie, Heinrich-Heine-Universität Düsseldorf, 40204 Düsseldorf, Germany

^b^Ernst Ruska-Zentrum für Mikroskopie und Spektroskopie mit Elektronen, Forschungszentrum Jülich GmbH, 52425 Jülich, Germany

Emails:

[lars.rademacher@hhu.de](mailto:lars.rademacher@hhu.de), [beglau@hhu.de](mailto:beglau@hhu.de), [heinent@hhu.de](mailto:heinent@hhu.de), [ju.barthel@fz-juelich.de](mailto:ju.barthel@fz-juelich.de), janiak@hhu.de

**Table of contents**

S1. Synthesis and Characterization of DCP-CFT600 and DCP- 2

S2. Characterization of Pd- and IrO_x_@CTF 10

S3. Comparison of NP@CTF materials 18

S4. References 23

# Synthesis and Characterization of DCP-CFT600 and DCP-

DCP-CTF600 and DCP-CTF750 were synthesized by the ionothermal method through mixing 0.300 g (2.3 mmol, 1 eq.) of the monomer 2,6-dicyanopyridine, DCP (2,6-pydridinedicarbonitrile) with 1.576 g (11.6 mmol; 5 eq.) zinc chloride, ZnCl_2_ in a molar ratio of 1:5 under argon (Ar) atmosphere, placed and sealed in an evacuated quartz glass ampoule. The ampoules were heated in a tube oven at 400 °C for 10 h and afterwards at 600 or 750 °C for 10 h yielding the corresponding CTFs (Scheme SI1). The product was stirred for several days in 100 mL water and in 100 mL 2 mol L^–1^ HCl. Subsequently the powder was washed with water (3x75 mL), tetrahydrofuran (3x75 mL) and acetone (3x75 mL). Afterwards, the black powders were dried under high vacuum (< 10^–6^ bar) for 16 h.

**Scheme S1.** Synthesis of DCP-CTF600/750 by the ionothermal method using 2,6-pyridinedicarbonitrile as monomer and zinc chloride functioning as ionic liquid, Lewis acid and porogen for the triazine ring and network formation.

Elemental analysis (EA) shows nitrogen contents in the materials comparable with the literature (Tuci et al., 2017; Artz et al., 2015; Sönmez et al., 2021). Measured values for the nitrogen content are below the theoretical values for the idealized structure. Nitrogen loss in the ionothermal synthesis of CTFs is well known and can be attributed to the high temperature during synthesis, resulting in elimination of H-C-N species. This leads to graphitization of the material and partial the loss of triazine functionalities (Tab. SI1) (Öztürk et al., 2020). Nevertheless, the nitrogen content is still high compared to other CTF materials, due to the additional pyridine nitrogen atom (Liu et al., 2019; Kuhn et al., 2008; Kuhn et al., 2009). The large difference to 100% for the sum of the CHN%, denoted as "rest" in Table S1, stems from an incomplete combustion of the samples during CHN analysis because thermogravimetric analysis up to 1000 °C shows the complete decomposition of the CTF material and thereby the absence of significant inorganic impurities (Fig. SI6).

**Table SI1.** Elemental analysis of DCP-CTF600 and DCP-CTF750.

| Material ^a^ | DCP/ZnCl_2_ molar ratio | Carbon (wt%) | Hydrogen (wt%) | Nitrogen (wt%) | Rest (wt%) |
| --- | --- | --- | --- | --- | --- |
| DCP-CTF calc. (C_7_H_3_N_3_) ^b^ |  | 65.11 | 2.34 | 32.55 | 0 |
| this work (400/600/3) ^c^ | 1:5 | 58.29 | 2.48 | 19.06 | 20.17 |
| this work (400/750/3) ^d^ | 1:5 | 78.54 | 0.91 | 9.56 | 10.99 |
| Tuci et al., 2017 (400/600/10) ^c^ | 1:5 | - | - | 21.6 | - |
| Tuci et al., 2017 (400/750/10) ^d^ | 1:5 | - | - | 7.4 | - |
| Artz et al., 2015 (400/600/10) ^c^ | 1:5 |  |  | 17.2 |  |
| Sönmez et al., 2021 (400/600/10) ^c^ | 1:5 | - | - | 18.8 |  |
| Kuhn et al., 2008 (400/-/10) ^e^ | 1:10 | 50.1 | 2.56 | 27.71 | 19.63 |

^a^ CTFs were heated to 400 °C for 10 h in a first step followed by a second step at 600 or 750 °C for 10 h (temperature step 1 / temperature step 2 / heating rate in °C/min to reach the temperature in step 1 and step 2). Kuhn et al. heated to 400 °C for 40 h only. ^b^ Calculation based on idealized structures of DCP-CTF with the identical elemental composition as the DCP monomer (C_7_H_3_N_3_).

^c^ DCP-CTF600

^d^ DCP-CTF750

^e^ DCP-CTF400

**Fig. SI1.** Powder X-ray diffraction (PXRD) patterns of the DCP-CTF600 and DCP-CTF750 show the absence of crystallinity, that is a rather amorphous material which is typical for CTFs synthesized by the ionothermal method (Tuci et al., 2017; Liu et al., 2019, Iwashita et al., 2004). Due to the high temperatures during ionothermal synthesis together with nitrogen loss, partial decomposition and defect formation in the structure a long-range order is missing in the CTF materials. Especially above 400 °C the CTF becomes mainly a nitrogen-doped carbon material (Öztürk et al., 2020). No crystalline ZnCl_2_ impurities were detected by PXRD.

The comparison of BET surface area and total pore volume from nitrogen sorption measurements in Table SI2 shows some minor differences. Noticeable, the BET surface of the CTF prepared at 400 °C by Kuhn et al. is significantly smaller, in line with the known strong increase of the porosity of the CTFs with temperature in the ionothermal synthesis (Tuci et al., 2017; Artz et al., 2015; Kuhn et al., 2008; Kuhn et al., 2009).


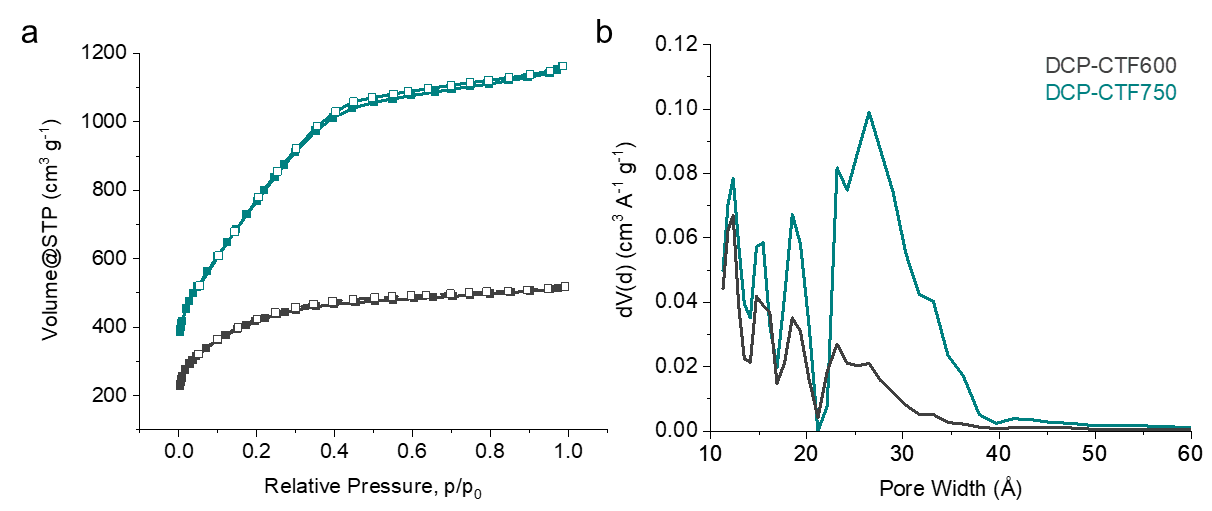


**Fig. SI2.** (a) Nitrogen sorption isotherms (adsorption: filled boxes; desorption: empty boxes) and (b) pore size distribution curves of pristine DCP-CTF600 and DCP-CTF750.

**Table SI2.** Comparison of BET surface areas and total pore volumes.

| Material ^a^ | Monomer/ZnCl_2_ molar ratio | BET surface area  (m^2^ g^–1^) | Total pore volume  (cm^3^ g^–1^) ^b^ |
| --- | --- | --- | --- |
| this work (400/600/3) ^c^ | 1:5 | 1334 | 0.79 |
| this work (400/750/3) ^d^ | 1:5 | 2542 | 1.77 |
| Tuci et al., 2017 (400/600/10) ^c^ | 1:5 | 1239 | 0.49 |
| Tuci et al., 2017 (400/750/10) ^d^ | 1:5 | 3040 | 1.51 |
| Artz et al., 2015 (400/600/10) ^c^ | 1:5 | 1179 | 0.64 |
| Sönmez et al., 2021 (400/600/10) ^c^ | 1:5 | 1050 | 0.57 |
| Kuhn et al., 2008 (400/-/10) ^e^ | 1:10 | 730 | 0.36 |

^a^ Listed CTFs were heated to 400°C in a pre-step followed by a second step at 600 or 750°C for 10 h (temperature step 1 / temperature step 2 / heating rate in °C/min to reach the temperature in step 1 and step 2). Kuhn et al. heated to 400 °C for 40 h only. ^b^ at p/p_0_ = 0.95 - 0.99

^c^ DCP-CTF600

^d^ DCP-CTF750

^e^ DCP-CTF400

X-ray photoelectron spectroscopy (XPS) of the neat CTFs (Fig. SI3) shows the formation of different nitrogen species typical for CTFs (Sönmez et al., 2021; Öztürk et al., 2020, Osadchii et al., 2017). Notably, the content of graphitic nitrogen at ~400 eV and oxidized nitrogen at ~402 eV depends on the synthesis temperature. High temperatures during synthesis promote an increased fraction of graphitic nitrogen, which is in good correlation with the improved electrocatalytic performance. The peaks at ~397 eV and 399 eV correspond to pyridinic and pyrrolic nitrogen. The high resolution spectra of the C 1s orbital confirm the presence of different carbon species (Sönmez et al., 2021; Öztürk et al., 2020, Liu et al., 2019). The peaks ~284, ~285 and ~287 eV belong to carbon bound to pyridinic/pyrrolic nitrogen and to the triazine-ring carbon atoms. The peak at ~290 eV is assigned to oxygen bonded carbon (Liu et al., 2020).


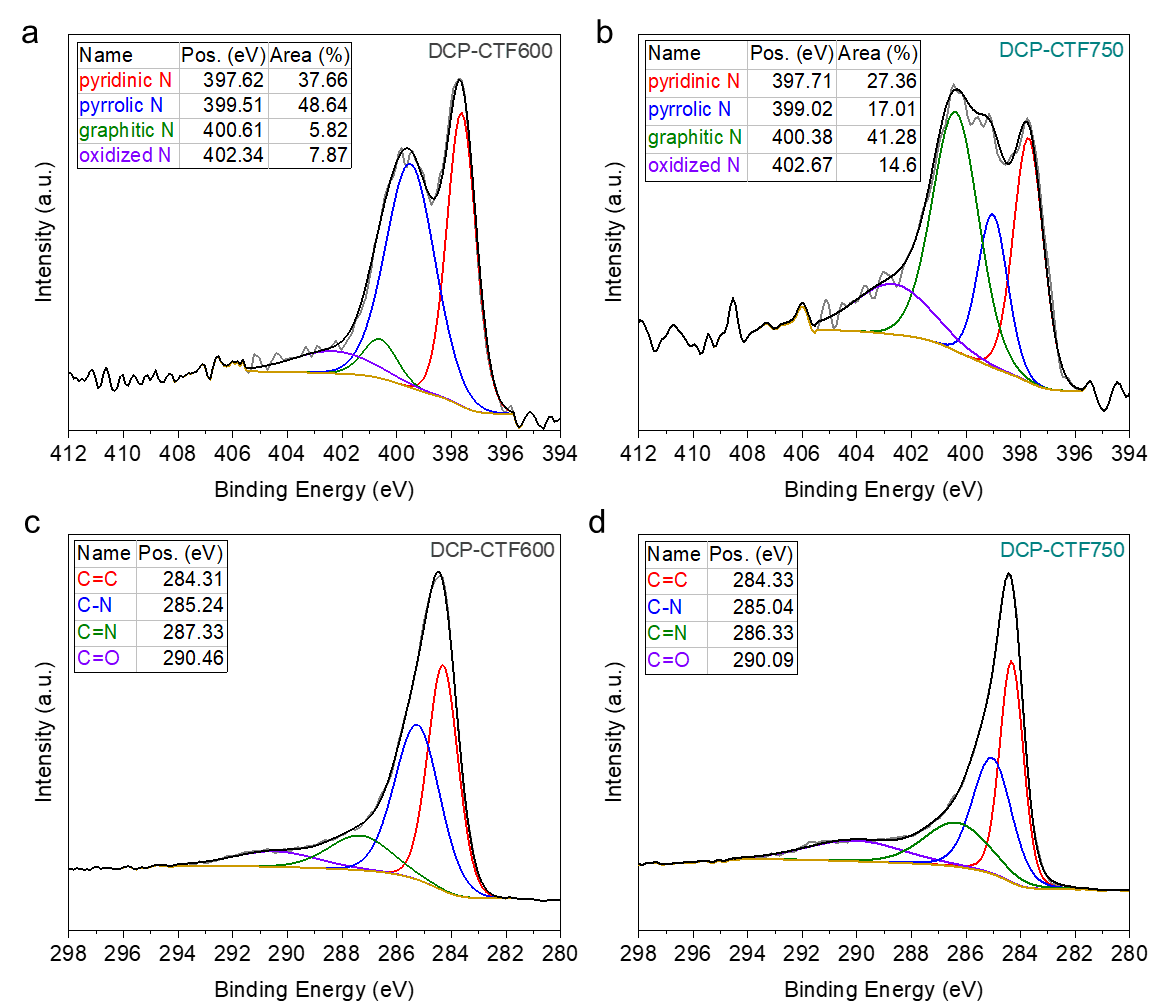


**Fig. SI3.** XPS measurements of (a, c) DCP-CTF600 and (b, d) DCP-CTF750 with (a, b) deconvoluted N 1s and (c, d) C 1s spectra.


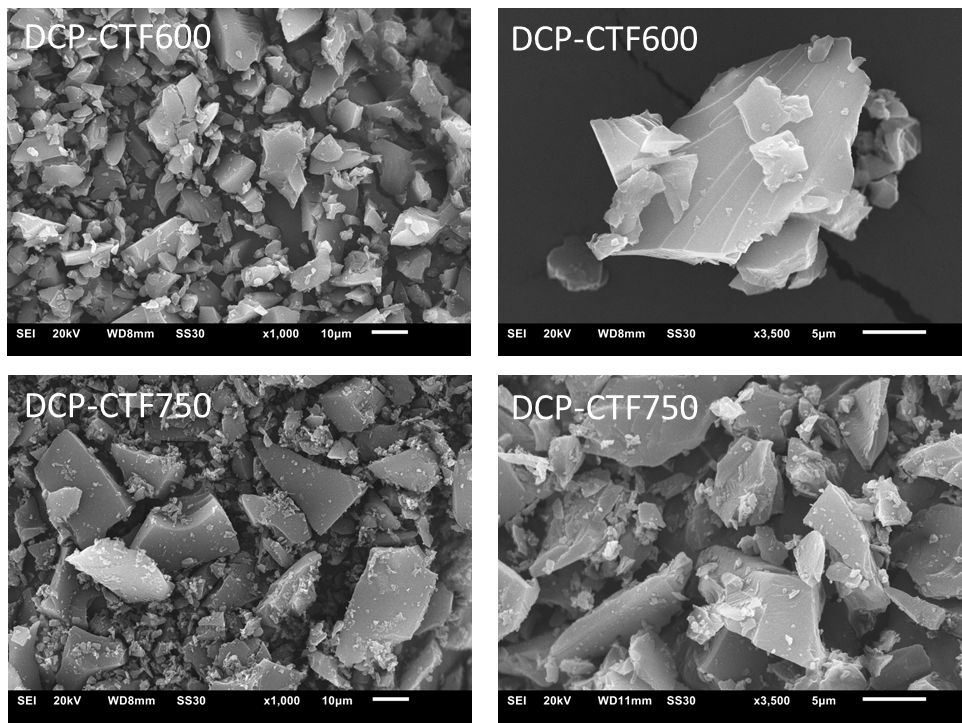


**Fig. SI4.** SEM images of DCP-CTF600 and DCP-CTF750.

Fourier transform infrared (FTIR) spectra of the DCP-CTFs demonstrate the disappearance of the DCP nitrile groups with their band at 2245 cm^–1^ and the formation of a triazine ring with its broad band at 1567 cm^–1^ (Fig. SI5).

**Fig. SI5.** Infrared spectra of the monomer 2,6-dicyanopyridine (DCP, 2,6-pyrdinedicarbonitrile), DCP-CTF600 and DCP-CTF750.

Fig. SI6 shows the thermogravimetric analysis (TGA) curves from the decomposition of the DCP-CTFs under synthetic air at a heating rate of 5 K min^–1^. Residual masses are 0.84 % for DCP-CTF600 and 1.08 % for DCP-CTF750 and appear to be due to an incomplete combustion as indicated by the still slightly negative slope at 1000 °C. Both CTFs demonstrate high thermal stability and start decomposing at 400 °C.

**Fig. SI6.** TGA curve for the decomposition of DCP-CTF600 and DCP-CTF750.

# Characterization of Pd- and IrO_x_@CTF

Fig. SI7 shows the survey spectra of Pd^29^@CTF750^IL^ and IrO_x_^14^@CTF750^PC^ with peaks for Pd, C, N and F (from IL) and Ir, C, N, O, respectively. Peaks belonging to oxygen are only detected in the Ir sample, while the Pd sample does not show any oxygen content. Thereby and by the deconvolution of the high-resolution spectra, XPS confirmed the presence of IrO_x_ and the absence of Ir(0) as in the Ir_4_(CO)_12_ precursor or in Ir metal (Fig. 2b,d; main text). Fluorine present in Pd^29^@CTF750^IL^ derives from residual IL.

The F KLL and O KLL peak are due the Auger electron in the Auger spectrum, which is part of the XP spectrum.


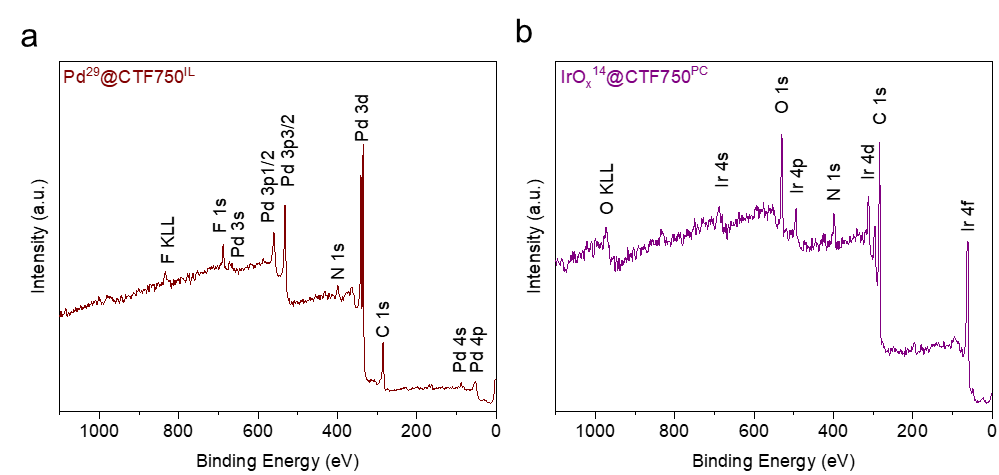


**Fig. SI7.** X-ray photoelectron survey spectra (XPS) of (a) Pd^29^@CTF750^IL^ and (b) IrO_x_^14^@CTF750^PC^.

Fig. SI8 displays additional transmission electron microscopy (TEM) images, particles size distributions and energy dispersive X-ray spectra (TEM-EDX) of the NP@CTF composites. Particles size distributions are based on 300 manually measured particles showing a Gaussian-similar distribution. Ir containing materials show very small nanoparticles, while Pd containing samples have average NP sizes from 11 to 13 nm which lie above the calculated crystallite size (from the Scherrer equation). Furthermore, for Pd@CTF, in particular, a shift towards larger particles sizes with higher metal amount can be observed. EDX in combination with TEM shows signals of Ir and Pd. Signals belonging to Si and Cu in the EDX spectra result from the used glass ware during synthesis or storage and from the carbon-coated copper TEM grid. Zn and Cl signals are not present in the EDX indicating the absence of ZnCl_2_.

**a)**


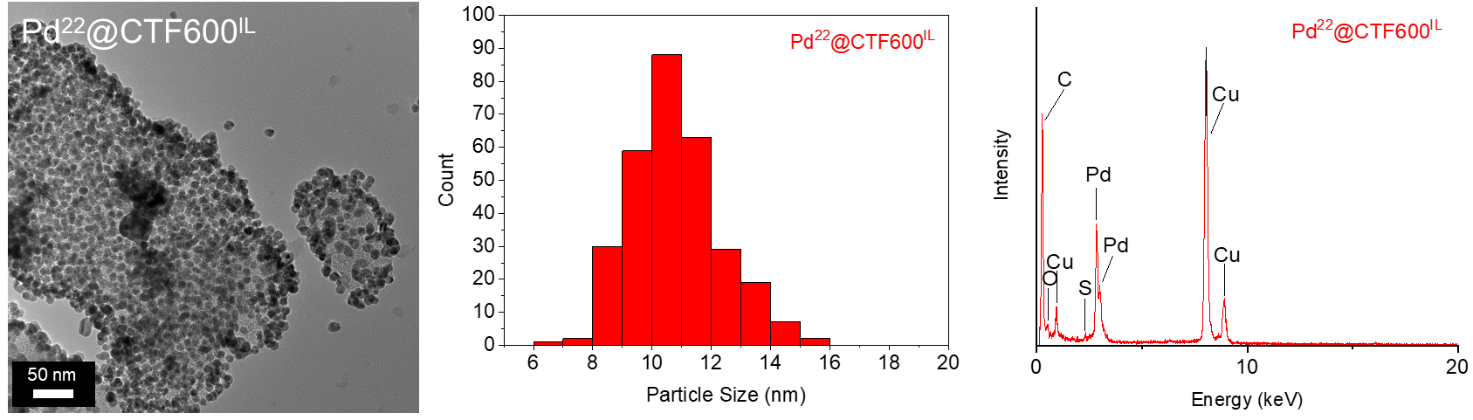


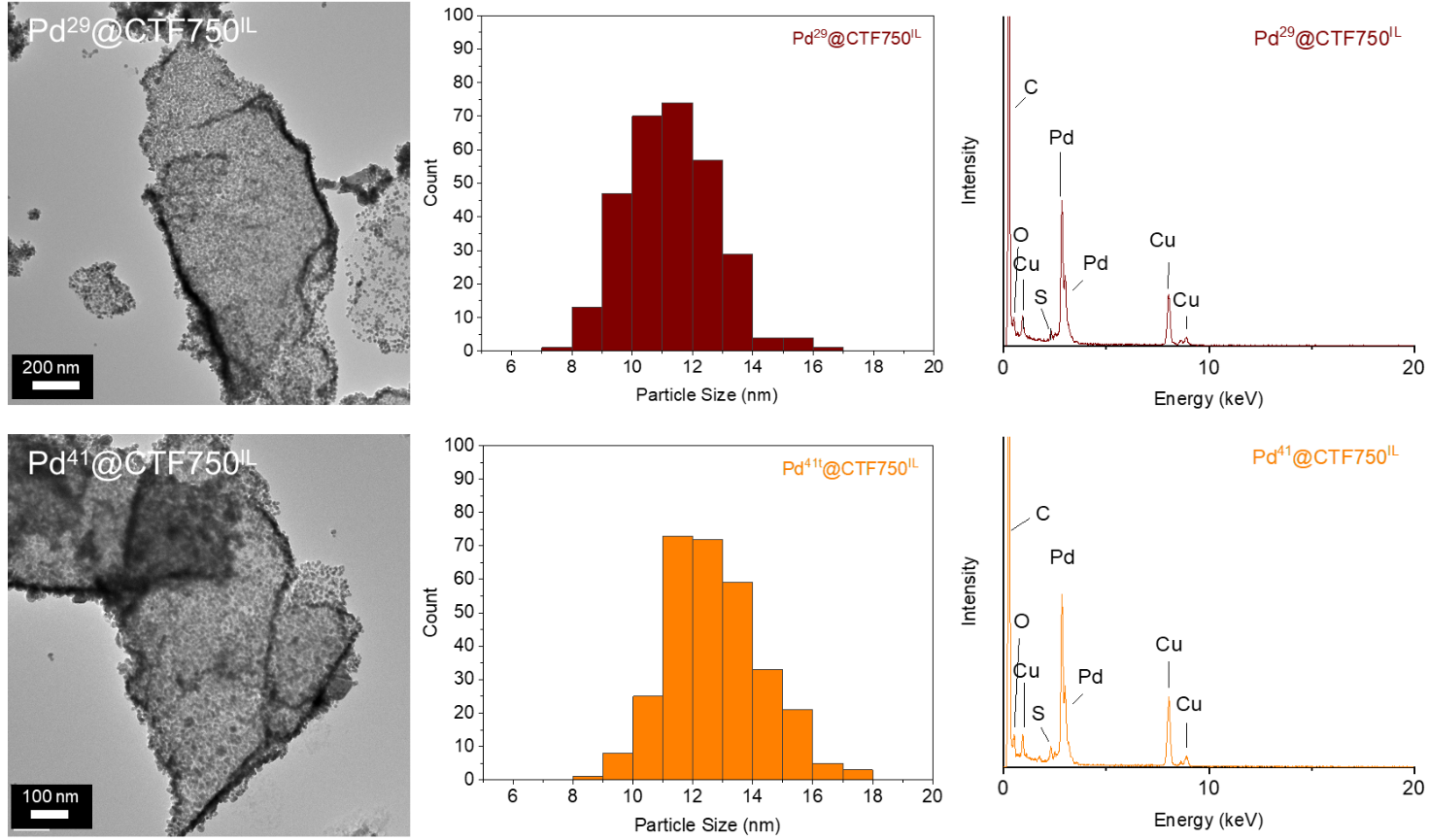


**b)**


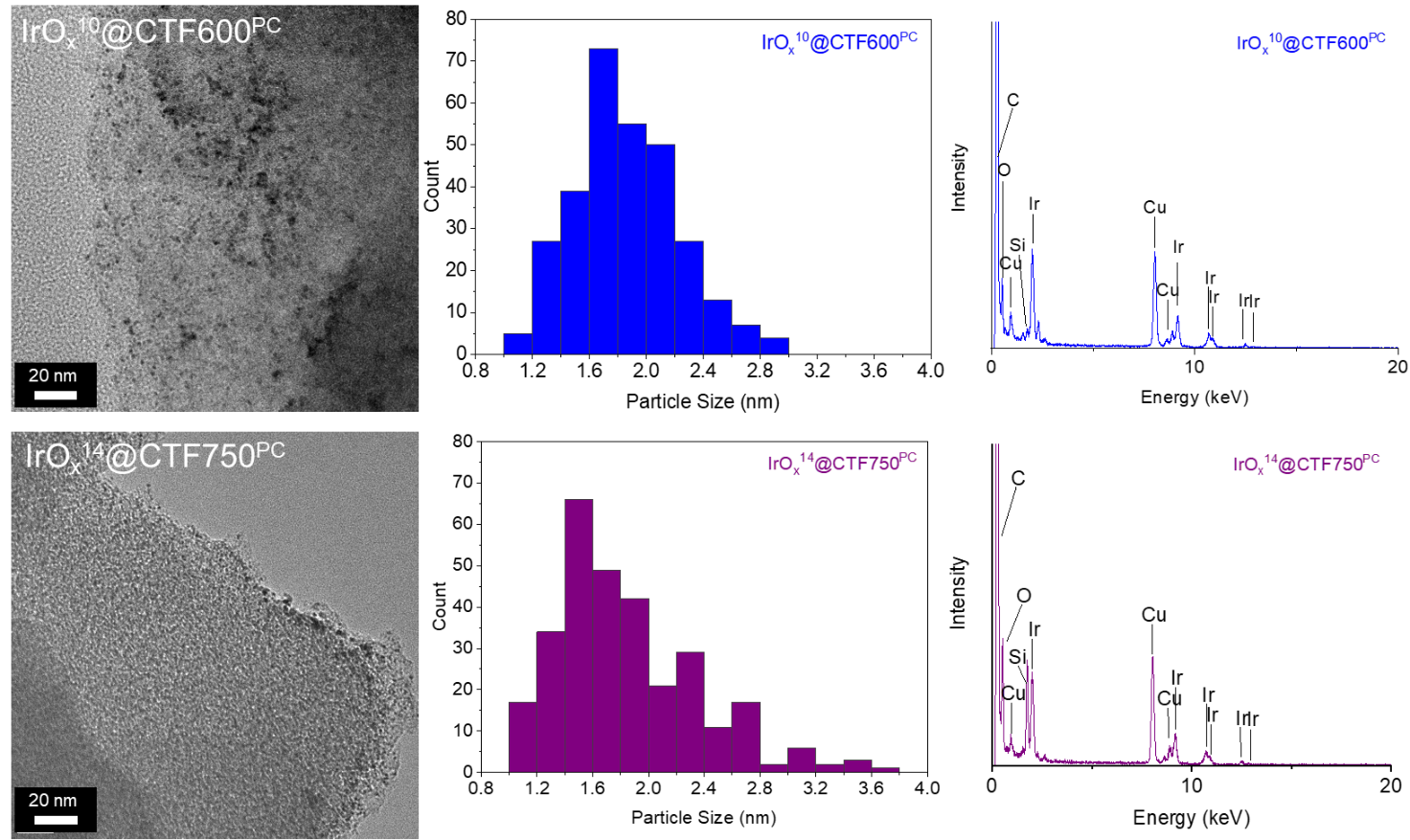


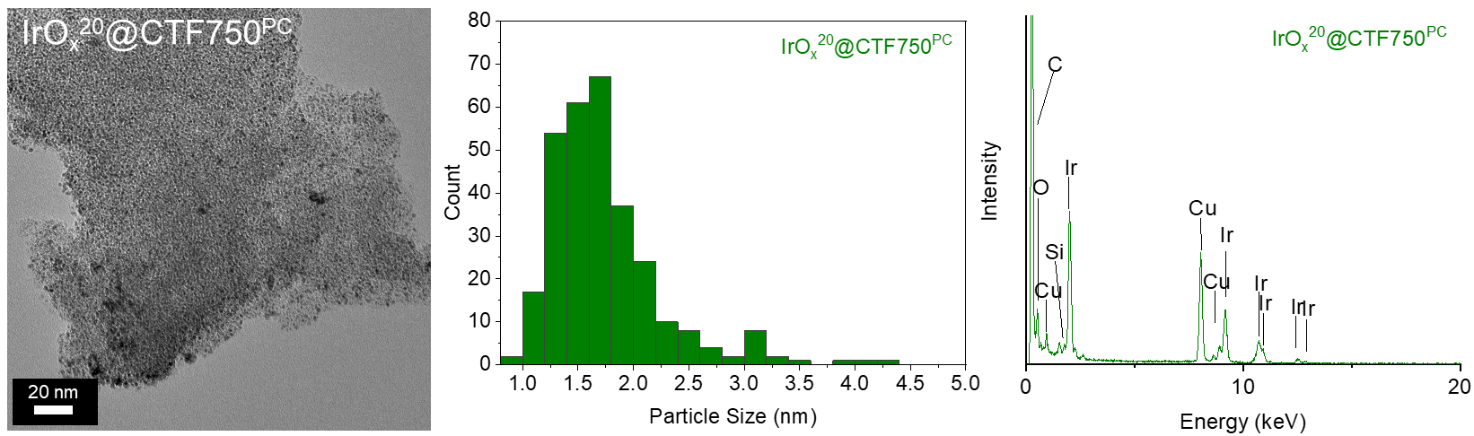


**Fig. SI8.** TEM pictures, particle size distribution and TEM-EDX spectra of (a) Pd@CTF and (b) IrO_x_@CTF samples.

Representative selected area electron diffraction (SAED) was conducted for Pd^29^@CTF750^IL^ and shows the characteristic ring pattern of face-centered cubic Pd (Fig. SI9) in accordance with the powder X-ray diffractograms (Fig. 1, main text) and the verification of Pd metal from XPS (Fig. 2, main text). SAED for IrO_x_ containing samples did not give reflections or a ring pattern due to the amorphous character together with the very small particle size.


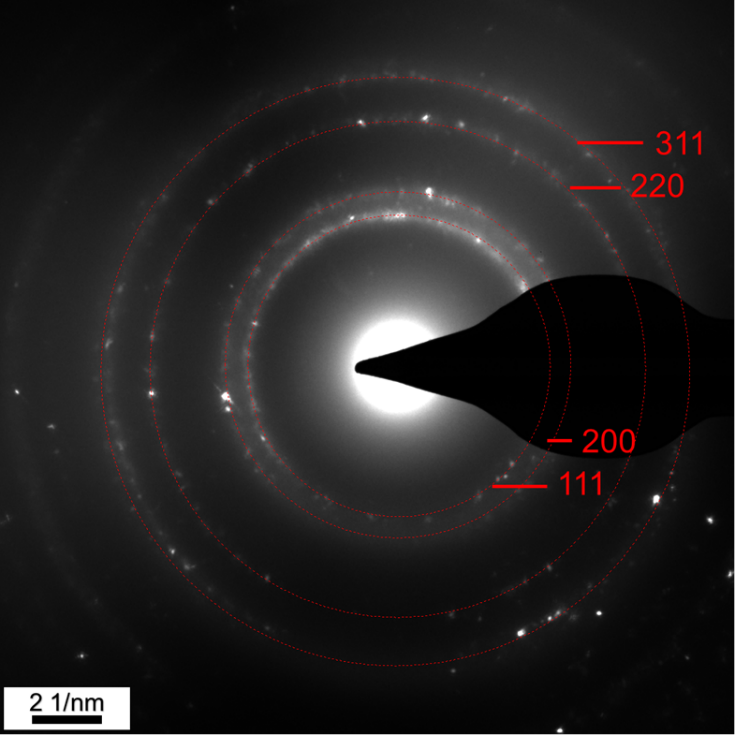


**Fig. SI9.** Exemplary selected area electron diffraction (SAED) of Pd^29^@CTF750^IL^ before electrocatalysis showing the ring pattern of *fcc*-Pd. The SAED of Pd^29^@CTF750^IL^ after the ORR stability test is given in Fig. SI11.

**Table SI3.** Experimental and expected BET surface areas of NP@CTF materials.

| Material | Experimentally measured BET surface area  (m^2^ g^–1^) | Expected BET surface area  (m^2^ g^–1^) ^a^ | Deviation (%) |
| --- | --- | --- | --- |
| Pd^22^@CTF600^IL^ | 904 | 1040 | -13 |
| Pd^29^@CTF750^IL^ | 1353 | 1805 | -25 |
| Pd^41^@CTF750^IL^ | 971 | 1500 | -35 |
| IrO_x_^10^@CTF600^PC^ | 1055 | 1201 | -12 |
| IrO_x_^14^@CTF750^PC^ | 1229 | 2186 | -44 |
| IrO_x_^20^@CTF750^PC^ | 918 | 2034 | -55 |

^a^ Calculation of expected BET by Eq. 1 (main text) based on the metal content determined by AAS

Fig. SI10 shows TEM images, particles size distributions and TEM-EDX spectra of IrO_x_^20^@CTF750^PC^ after the HER stability test and of Pd^29^@CTF750^IL^ after the ORR stability test. The presence of Ir and Pd after 1000 CVs in both materials remains unchanged. The particle size distribution shows a negligible shift to larger particle sizes for the IrO_x_ sample with a constant average size of 2 ± 1 nm and a small shift for Pd from 11 ± 2 to 14 ± 3 after the CV stability test.


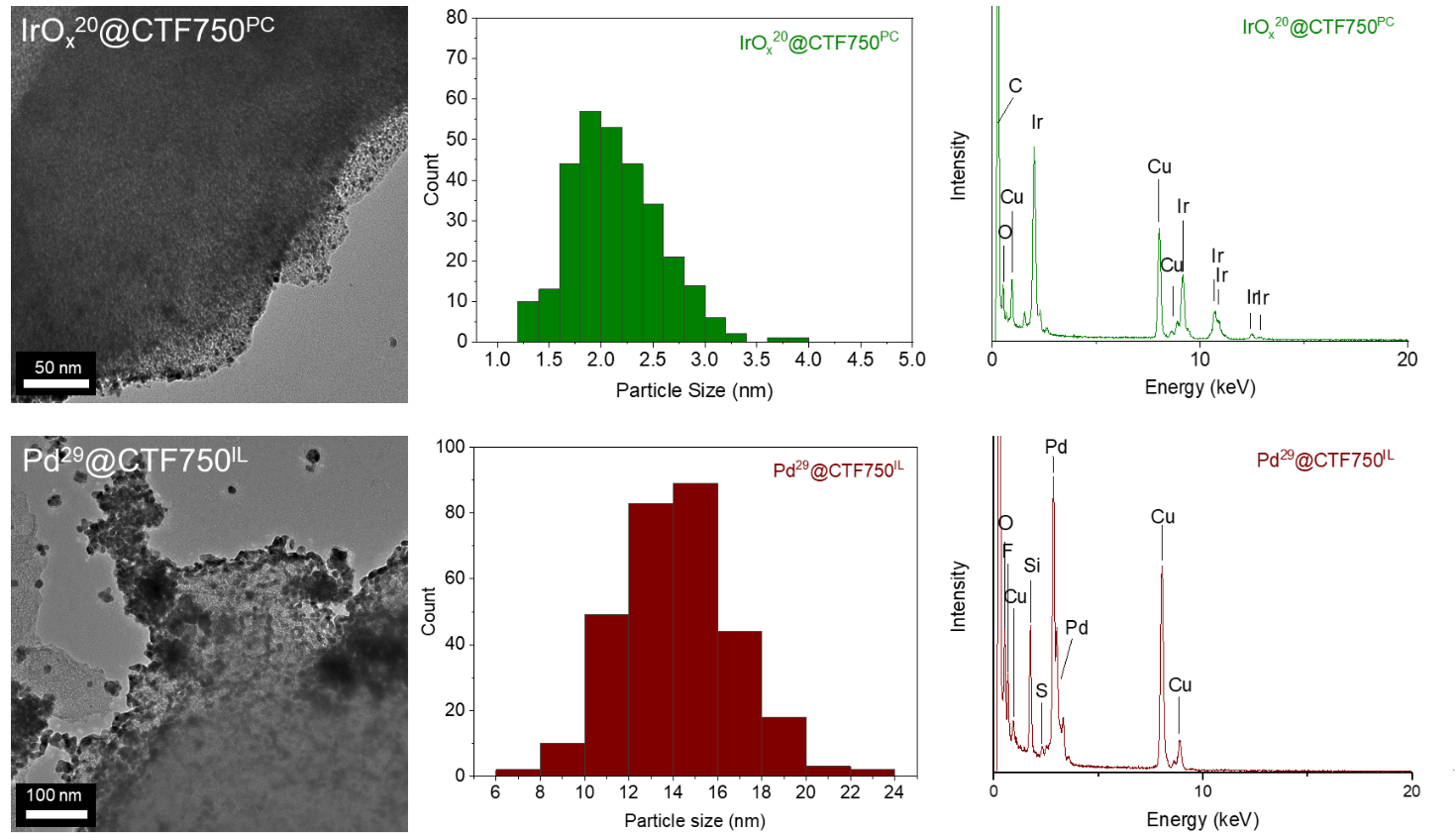


**Fig. SI10.** TEM images, particles size distribution and TEM-EDX spectrum of IrO_x_^20^@CTF750^PC^ (HER) and Pd^29^@CTF750^IL^ (ORR) after durability test.


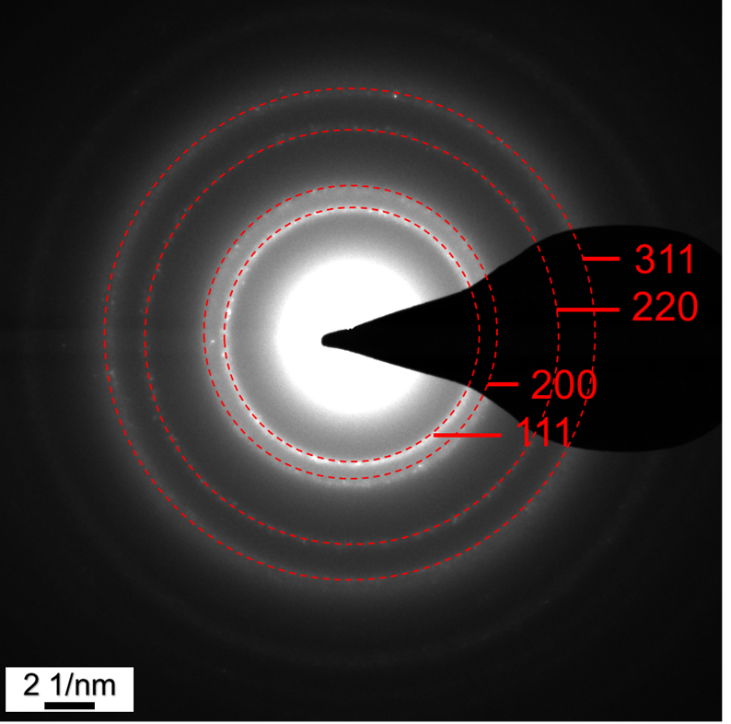


**Fig. SI11.** SAED of Pd^29^@CTF750^IL^ after ORR stability test with 1000 CVs showing the ring pattern of *fcc*-Pd.

Fig. SI12 and Table SI3 summarize the results from electrochemical impedance spectroscopy (EIS) of the NP@CTF materials performed under HER and ORR conditions. Data were plotted in a Nyquist plot and fitted to Voigt circuit models (Fig. SI12) to obtain quantitative data for the charge transfer resistances (Shi et al., 2016; Ipadeola and Ozoemena, 2020; Ruiz-Camacho et al., 2017). The curvature of the Nyquist plots is determined by the applied potential, the electrolyte and the properties of the electrocatalyst. Under HER conditions the radii of the semicircles and the calculated charge transfer resistances significantly decrease with the decrease in overpotential (Fig. SI12a) which suggests lower charge transfer resistances following the same trend like in the measured overpotentials (Table 2, main text). Under ORR conditions the samples behave different. Except for Pd^29^@DCP-CTF750 the charge transfer resistance of the other samples exceeds 100,000 Ω.


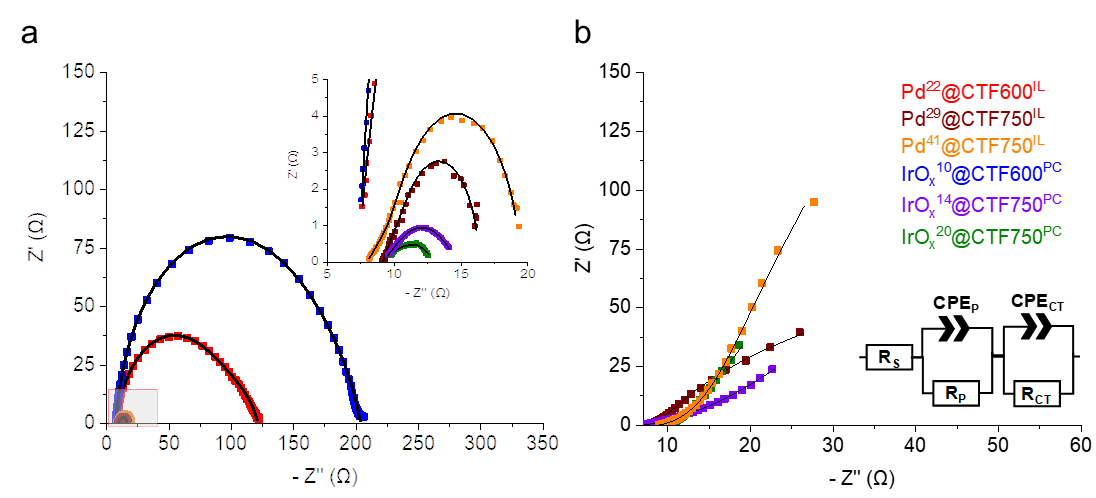


**Fig. 12.** Nyquist plots of Pd- and IrO_X_@CTF materials (a) in 0.5 mol L^–1^ H_2_SO_4_ and (b) 1.0 mol L^–1^ KOH. Data were fitted to a Voigt circuit model (solid lines) with resistance, R, and constant phase, CPE, elements for the electrolyte, the electrode porosity and the charge transfer.

**Table SI4.** Charge transfer resistance of Pd- and IrO_X_@CTF materials for HER and ORR conditions.

| Material | R_CT_ in 0.5 mol L^–1^ H_2_SO_4_ (Ω) | R_CT_ in 1.0 mol L^–1^ KOH (Ω) |
| --- | --- | --- |
| Pd^22^@CTF600^IL^ | 38.4 | - |
| Pd^29^@CTF750^IL^ | 4.6 | 279.8 |
| Pd^41^@CTF750^IL^ | 6.4 | > 100,000 |
| IrO_x_^10^@CTF600^PC^ | 55.8 | - |
| IrO_x_^14^@CTF750^PC^ | 4.1 | > 100,000 |
| IrO_x_^20^@CTF750^PC^ | 2.3 | > 100,000 |

# Comparison of NP@CTF materials

**Table SI5.** Comparison of NP@CTF materials towards HER.

| **Material** | **Synthesis** | **Metal content (wt%)** | **Setup, electrolyte and catalyst loading** | **Overpotential at 10 mA cm^-2^**  **(mV)** | **Tafel slope**  **(mV dec^-1^)** | **Lit.** |
| --- | --- | --- | --- | --- | --- | --- |
| Pd@DCP-CTF | Microwave reaction in [BMIm][NTf_2_] using Pd(acac)_2_ | 22 - 41 wt% (by AAS) | RDE, 0.5 mol L^–1^ H_2_SO_4_, 0.225 mg cm^–2^ | 135 - 325 | 78 - 166 | **This work** |
| IrO_x_@DCP-CTF | Microwave reaction in PC using Ir_4_(CO)_12_ | 10 - 20 wt% (by AAS) | RDE, 0.5 mol L^–1^ H_2_SO_4_, 0.225 mg cm^–2^ | 47 - 368 | 28 - 132 | **This work** |
| Rh/CTF-1 | Microwave reaction in [BMIm][NTf_2_] using Rh_6_(CO)_16_ | 4.4 wt% (by AAS) | RDE, 0.5 mol L^–1^ H_2_SO_4_, 0.015 mg cm^–2^ (metal content) | 58 | 37 | Siebels et al., 2019 |
| Pt/CTF-1 | Microwave reaction in [BMIm][NTf_2_] using Pt(acac)_2_ | not given | RDE, 0.5 mol L^–1^ H_2_SO_4_, 0.015 mg cm^–2^ (metal content) | 111 | 88 | Siebels et al., 2019 |
| CTF@MoS_2_-5 ^a^ | Hydrothermal reaction using Thiourea and (NH_4_)_6_Mo_7_O_24_ | not given | GC, 0.5 mol L^–1^ H_2_SO_4_, 0.159 mg cm^–2^ | 93 | 43 | Qiao et al., 2019 |
| DCP-CTF@Pd-MC | Decomposition of Pd(OAc)_2_ in CH_2_Cl_2_ | not given | GC, 0.5 mol L^–1^ H_2_SO_4_, 0.159 mg cm^–2^ | 71 | 45.6 | Zhang et al., 2020 |
| DCP-CTF@Pt-MC | Chemical reduction of H_2_PtCl_6_ using NaBH_4_ in H_2_O | not given | GC, 0.5 mol L^–1^ H_2_SO_4_, 0.159 mg cm^–2^ | 60 | 30.7 | Zhang et al., 2020 |
| n-Pd@NDCDs ^b^ | Hydrothermal | not given | GC, 0.5 mol L^–1^ H_2_SO_4_, 0.05 mg cm^–2^ | 291 | 135 | Chandrasekaran et al., 2020 |
| Pd_120_/Ti ^c^ | Aerosol assisted chemical vapour deposition (AACVD) | - | 0.5 mol L^–1^ H_2_SO_4_ | 40 | 52.3 | Ehsan et al., 2020 |
| PdMnCo/NC-2 ^d^ | Sonication | 26.1 wt% (Pd, by ICP) | GC, 0.5 mol L^–1^ H_2_SO_4_, 0.285 mg cm^–2^ | 34 | 31 | Zhang et al., 2017 |
| Pd/C | *Commercial (Sigma Aldrich)* | 30 wt% | GC, 0.5 mol L^–1^ H_2_SO_4_, 0.285 mg cm^–2^ | 96 | 43 | Zhang et al., 2017 |
| Pd_83.5_Ir_12.6_ | Wet-chemical method | - | GC, 0.5 mol L^–1^ H_2_SO_4_, 0.103 mg cm^–2^ | 73 | 48.4 | Wang et al., 2020 |
| Ir/SiNW ^e^ | Hydrothermal reaction using H_2_IrCl_6_ | 17.7 (by XRF) | GC, 0.5 mol L^–1^ H_2_SO_4_, 0.339 mg cm^–2^ | 22 | 20 | Sheng et al., 2019 |
| G-Ir | Thermal reduction of (NH_4_)_2_[IrCl_6_] under H_2_ atmosphere | 1.48 (by EDS) | GC, 0.5 mol L^–1^ H_2_SO_4_ | ~ 280 | 40 | Lim et al., 2015 |
| IrO_2_/N@C ^f^ | Thermal treatment | not given | Ti foil, 0.5 mol L^–1^ HClO_4_ | 35 | 31 | Kundu et al., 2020 |
| IrO_2_ | Commercial | - | Ti foil, 0.5 mol L^–1^ HClO_4_ | 145 | 49 | Kundu et al., 2020 |

^a^ CTF-1 is used. ^b^ Pd nanoparticles supported nitrogen doped carbon dots. ^c^ Pd film grown on Ti foil with 120 min deposition time. ^d^ PdMnCo nanoparticles on nitrogen doped carbon. ^e^ Iridium/silicon nanowire composite. ^f^ Irdium oxide on nitrogen doped carbon.

**Table SI6.** Comparison of NP@CTF materials towards ORR.

| **Material** | **Synthesis** | **Metal content (wt%)** | **Setup, electrolyte and catalyst loading** | **Half-wave potential**  **(mV)** | **Current density at 0.4 V (mA cm**^–^**^2^)** | **Tafel slope**  **(mV dec**^–^**^1^)** | **Lit.** |
| --- | --- | --- | --- | --- | --- | --- | --- |
| Pd@DCP-CTF | Microwave reaction in [BMIm][NTf_2_] using Pd(acac)_2_ | 22 - 41 wt% (by AAS) | RDE, 1.0 mol L^–1^ KOH, 0.225 mg cm^–2^ | 810 - 872 | 3.1 - 3.8 | 58 | **This work** |
| IrO_x_@DCP-CTF | Microwave reaction in PC using Ir_4_(CO)_12_ | 10 - 20 wt% (by AAS) | RDE, 1.0 mol L^–1^ KOH, 0.225 mg cm^–2^ | 823 - 828 | 3.8 - 4.1 | 56 - 90 | **This work** |
| Pd/FLG ^a^ | Thermal reduction of Pd(NO_3_)_2_ under H_2_ atmosphere | 40 wt%  (by TGA) | (R)RDE, 0.1 mol L^–1^ KOH, 25 µg cm^–2^ | 955 | ~ 5.5 | 32 | Truong-Phuoc et al., 2014 |
| Pd/C | Thermal reduction of Pd(NO_3_)_2_ under H_2_ atmosphere | 40 wt%  (by TGA) | (R)RDE, 0.1 mol L^–1^ KOH, 25 µg cm^–2^ | 905 | ~ 5.5 | 62 | Truong-Phuoc et al., 2014 |
| Ir-SAC ^b^ | Decomposition of Ir-ZIF-8 | 0.2 (by ICP) | (R)RDE, 0.1 mol L^–1^ HClO_4_, 0.4 mg cm^–2^ | 864 | ~ 6.0 | 41 | Xiao et al., 2019 |
| Ir/C | Commercial | 20 | (R)RDE, 0.1 mol L^–1^ HClO_4_, 0.036 mg cm^–2^ | 590 | - | 97 | Xiao et al., 2019 |
| Mn_0.8_-Fe_0.2_ LDH ^c^ | Co-participation | - | RDE, 1.0 mol L^–1^ KOH, 200 µg cm^–2^ | - | ~ 2.2 | - | Iwai et al., 2019 |
| Pt/C | Commercial | not given | RDE, 1.0 mol L^–1^ KOH, 200 µg cm^–2^ | - | ~ 2.7 | - | Iwai et al., 2019 |
| Ni/CTF-1 | Microwave reaction in [BMIm][NTf_2_] using Ni(COD)_2_ | 22 wt% (by AAS) | RDE, 1.0 mol L^–1^ KOH, 0.225 mg cm^–2^ | 775 | ~ 4.0 | not given | Öztürk et al., 2020 |
| Cu-CTF/CP ^d^ | Impregnation of CuCl_2_ | 0.58 at%  (by XPS) | (R)RDE, 0.1 mol L^–1^ NaOH, 0.24 mg cm^–2^ | 810 | ~ 4.5 | not given | Iwase et al., 2015 |
| Co-CTF/KB ^d^ | Impregnation of Co(OAc)_2_ | 4 wt% | (R)RDE; 0.1 mol L^–1^ KOH, 0.255 mg cm^–2^ | 830 | ~ 6.0 | 65 | Zhou et al., 2021 |

^a^ Few-layered graphene supported Pd. ^b^ Iridium as single-atom catalyst. ^c^ Layered double hydroxide. ^d^ DCP based CTF.

# References

Tuci, G., Pilaski, M., Ba, H., Rossin, A., Luconi, L., Caporali, S., Pham-Huu, C., Palkovits, R., Giambastiani, G. (2017). Unraveling surface basicity and bulk morphology relationship on covalent triazine frameworks with unique catalytic and gas adsorption properties. *Adv. Funct. Mater.* 27, 1605672. doi: 10.1002/adfm.201605672

Artz, J., Mallmann, S., Palkovits, R. (2015). Selective aerobic oxidation of HMF to 2,5‐diformylfuran on covalent triazine frameworks‐supported Ru catalysts. *ChemSusChem* 8, 672–679. doi: 10.1002/cssc.201403078

Sönmez, T., Belthle, K. S., Iemhoff, A., Uecker, J., Artz, J., Bisswanger, T., Stampfer, C., Hamzah, H. H., Nicolae, S. A., Titirici, M.-M., Palkovits, R. (2021). Metal free-covalent triazine frameworks as oxygen reduction reaction catalysts–structure–electrochemical activity relationship. *Catal. Sci. Technol.* 11, 6191–6204. doi: 10.1039/D1CY00405K

Öztürk, S., Xiao, Y. X., Dietrich, D., Giesen, B., Barthel, J., Ying, Yang, X.-Y., Janiak, C. (2020). Nickel nanoparticles supported on a covalent triazine framework as electrocatalyst for oxygen evolution reaction and oxygen reduction reactions. *Beilstein J. Nanotechnol.* 11*,* 770–781. doi: 10.3762/bjnano.11.62

Liu, M., Guo, L., Jin, S., Tan, B. (2019). Covalent triazine frameworks: synthesis and applications *J. Mater. Chem*. *A* 7, 5153–5172. doi: 10.1039/C8TA12442F

Kuhn, P., Antonietti, M., Thomas, A. (2008). Porous, covalent triazine‐based frameworks prepared by ionothermal synthesis. *Angew. Chem. Int. Ed.* 47, 3450–3453. doi: 10.1002/anie.200705710

Kuhn, P., Thomas, A., Antonietti, M. (2009). Toward Tailorable Porous Organic Polymer Networks: A High-Temperature Dynamic Polymerization Scheme Based on Aromatic Nitriles. *Macromolecules* 42, 319–326. doi: 10.1021/ma802322j

Iwashita, N., Park, C. R., Hiroyuki, F., Shiraishi, M., Inagaki, M. (2004). Specification for a standard procedure of X-ray diffraction measurements on carbon materials. *Carbon* 42, 701-714. doi: 10.1016/j.carbon.2004.02.008

Osadchii, D. Y., Olivos-Suarez, A. I., Bavykina, A. V., Gascon, J. (2017). Revisiting Nitrogen Species in Covalent Triazine Frameworks. *Langmuir* 33, 14278−14285. doi: 10.1021/acs.langmuir.7b02929

Liu, Q., Yang, S., Repich, H., Zhai, Y., Xu, X., Liang, Y., Li, H., Wang, H., Yu, F. (2020). Porous Functionalized Covalent-Triazine Frameworks for Enhanced Adsorption Toward Polysulfides in Li-S Batteries and Organic Dyes. *Front. Chem.* 8, 584204. doi: 10.3389/fchem.2020.584204

Shi, Z., Wang, Y., Lin, H., Zhang, H., Shen, M., Xie, S., Zhang, Y., Gao, Q., Tang, Y. (2016) Porous nanoMoC@graphite shell derived from a MOFs-directed strategy: an efficient electrocatalyst for the hydrogen evolution reaction. *J. Mater. Chem.* 4, 6006–6013. doi: 10.1039/C6TA01900E

Ipadeola, A. K. and Ozoemena, K. I. (2020). Alkaline water-splitting reactions over Pd/Co-MOF-derived carbon obtained via microwave-assisted synthesis. *RSC Adv.* 10, 17359–17368. doi: 10.1039/D0RA02307H

Ruiz-Camacho, B., Vera, J. B., Medina-Ramírez, A., Fuentes-Ramírez, R., Carreño-Aguilera, G. (2017). EIS analysis of oxygen reduction reaction of Pt supported on different substrates. *Int. J. Hydrog. Energy* 42, 30364–30373. doi: 10.1016/j.ijhydene.2017.08.087

Siebels, M., Schlüsener, C., Thomas, J., Xiao, Y. X., Yang, X. Y., Janiak, C. (2019). Rhodium nanoparticles supported on covalent triazine-based frameworks as re-usable catalyst for benzene hydrogenation and hydrogen evolution reaction. *J. Mater. Chem. A* 7, 11934–11943. doi: 10.1039/C8TA12353E

Qiao, S., Zhang, B., Li, Q., Li, Z., Wang, W., Zhao, J., Zhang, X., Hu, Y. (2019). Pore Surface Engineering of Covalent Triazine Frameworks@MoS2 Electrocatalyst for the Hydrogen Evolution Reaction. *Chem. Sus. Chem.* 12, 5032–5040. doi: 10.1002/cssc.201902582

Zhang, B., Zhang, Y., Hou, M., Wang, W., Hu, S., Cen, W., Cao, X., Qiao, S., Han, B.-H. (2021). Pristine, metal ion and metal cluster modified conjugated triazine frameworks as electrocatalysts for hydrogen evolution reaction. *J. Mater. Chem. A* 9, 10146–10159. doi: 10.1039/D1TA00589H

Chandrasekaran, P., Edison, T. N. J. I., Sethuraman, M. G. (2020). Electrocatalytic performance of carbon dots/palladium nanoparticles composite towards hydrogen evolution reaction in acid medium. *Int. J. Hydrog. Energy* 45, 53, 28800–28811. doi: 10.1016/j.ijhydene.2020.07.262

Ehsan, M. A., Suliman, M. H., Rehman, A., Hakeem, A. S., Yamani, Z. H., Qamar, M. (2020). Direct deposition of a nanoporous palladium electrocatalyst for efficient hydrogen evolution reaction. *New J. Chem.* **44**, 7795–7801. doi: 10.1039/D0NJ00507J

Zhang, R., Sun, Z., Feng, R., Lin, Z., Liu, H., Li, M., Yang, Y., Shi, R., Zhang, W., Chen, Q. (2017). Rapid Adsorption Enables Interface Engineering of PdMnCo Alloy/Nitrogen-Doped Carbon as Highly Efficient Electrocatalysts for Hydrogen Evolution Reaction. *ACS Appl. Mater. Interfaces* 9, 38419–38427. doi: 10.1021/acsami.7b10016

Wang, C., Xu, H., Shang, H., Jin, L., Chen, C., Wang, Y., Yuan, M., Du, Y. (2020). Ir-Doped Pd Nanosheet Assemblies as Bifunctional Electrocatalysts for Advanced Hydrogen Evolution Reaction and Liquid Fuel Electrocatalysis. *Inorg. Chem.* 59, 3321–3329. doi: 10.1021/acs.inorgchem.0c00132

Sheng, M., Jiang, B., Wu, B., Liao, F., Fan, X., Lin, H., Li, Y., Lifshitz, Y., Lee, S.-T., Shao, M. (2019). Approaching the Volcano Top: Iridium/Silicon Nanocomposites as Efficient Electrocatalysts for the Hydrogen Evolution Reaction. *ACS Nano* 13, 2786–2794. doi: 10.1021/acsnano.8b07572

Lim, C. S., Sofer, Z., Toh, R. J., Eng, A. Y. S., Luxa, J., Pumera, M. (2015). Iridium- and Osmium-decorated Reduced Graphenes as Promising Catalysts for Hydrogen Evolution. *ChemPhysChem* 16, 1898–1905. doi: 10.1002/cphc.201500174

Kundu, M. K., Mishra, R., Bhowmik, T., Kanrar, S., Barman, S. (2020). Three-dimensional hierarchically porous iridium oxide-nitrogen doped carbon hybrid: An efficient bifunctional catalyst for oxygen evolution and hydrogen evolution reaction in acid. *Int. J. Hydrog. Energy* 44, 6036–6046. doi: 10.1016/j.ijhydene.2019.12.186

Truong-Phuoc, L., Pham-Huu, C., Da Costa, V., Janowska, I. (2014). Few-layered graphene-supported palladium as a highly efficient catalyst in oxygen reduction reaction. ***Chem. Commun.*** **50**, 14433–14435. doi: 10.1039/C4CC05527F

Xiao, M., Zhu, J., Li, G., Li, N., Li, S., Cano, Z. P., Ma, L., Cui, P., Xu, P., Jiang, G., Jin, H., Wang, S., Wu, T., Lu, J., Yu, A., Su, D., Chen, Z. (2019). A Single-Atom Iridium Heterogeneous Catalyst in Oxygen Reduction Reaction. *Angew.* *Chem. Int. Ed.* 58, 9640–9645. doi: 10.1002/anie.201905241

Iwai, Y., Miura, A., Rosero-Navarro, N. C., Higuchi, M., Tadanaga, K. (2019). Composition, valence and oxygen reduction reaction activity of Mn-based layered double hydroxides. *J. Asian Ceram. Soc.* 7, 147–153. doi: 10.1080/21870764.2019.1581321

Iwase, K., Yoshioka, T., Nakanishi, S., Hashimoto, K., Kamiya, K. (2015). Copper‐modified covalent triazine frameworks as non‐noble‐metal electrocatalysts for oxygen reduction. *Angew. Chem. Int. Ed.* 54, 11068–11072. doi: 10.1002/anie.201503637

Zhou, S., Xiao, Z., Yang, Q., Huang, X., Niu, Y., Ma, Y., Zhi, L. (2021). Atomic cobalt anchored on covalent triazine frameworks with ultra-high performance towards oxygen reduction reaction. *Sci. China Mater.* 64, 2221–2229. doi: 10.1007/s40843-020-1609-9
